# Genome size and endoreplication in two pairs of cytogenetically contrasting species of *Pulmonaria* (Boraginaceae) in Central Europe

**DOI:** 10.1093/aobpla/plac036

**Published:** 2022-08-18

**Authors:** Lukáš Koprivý, Viera Fráková, Vladislav Kolarčik, Lenka Mártonfiová, Matej Dudáš, Pavol Mártonfi

**Affiliations:** Institute of Biology and Ecology, Faculty of Science, Pavol Jozef Šafárik University, Mánesova 23, SK-041 54 Košice, Slovak Republic; Botanical Garden, Pavol Jozef Šafárik University, Mánesova 23, SK-043 52 Košice, Slovak Republic; Institute of Biology and Ecology, Faculty of Science, Pavol Jozef Šafárik University, Mánesova 23, SK-041 54 Košice, Slovak Republic; Institute of Biology and Ecology, Faculty of Science, Pavol Jozef Šafárik University, Mánesova 23, SK-041 54 Košice, Slovak Republic; Botanical Garden, Pavol Jozef Šafárik University, Mánesova 23, SK-043 52 Košice, Slovak Republic; Institute of Biology and Ecology, Faculty of Science, Pavol Jozef Šafárik University, Mánesova 23, SK-041 54 Košice, Slovak Republic; Institute of Biology and Ecology, Faculty of Science, Pavol Jozef Šafárik University, Mánesova 23, SK-041 54 Košice, Slovak Republic; Botanical Garden, Pavol Jozef Šafárik University, Mánesova 23, SK-043 52 Košice, Slovak Republic

**Keywords:** Boraginaceae, endoreplication, flow cytometry, genome size, geophytes, *Pulmonaria*

## Abstract

Genome size is species-specific feature and commonly constant in an organism. In various plants, DNA content in cell nucleus is commonly increased in process of endoreplication, cellular-specific multiplication of DNA content without mitosis. This leads to the endopolyploidy, the presence of multiplied chromosome sets in a subset of cells. The relationship of endopolyploidy to species-specific genome size is rarely analysed and is not fully understood. While negative correlation between genome size and endopolyploidy level is supposed, this is species- and lineage-specific. In the present study, we shed light on this topic, exploring both genome size and endoreplication-induced DNA content variation in two pairs of morphologically similar species of *Pulmonaria*, *P. obscura*–*P. officinalis* and *P. mollis*–*P. murinii*. We aim (i) to characterize genome size and chromosome numbers in these species using cytogenetic, root-tip squashing and flow cytometry (FCM) techniques; (ii) to investigate the degree of endopolyploidy in various plant organs, including the root, stem, leaf, calyx and corolla using FCM; and (iii) to comprehensively characterize and compare the level of endopolyploidy and DNA content in various organs of all four species in relation to species systematic relationships and genome size variation. We have confirmed the diploid–dysploid nature of chromosome complements, and divergent genome sizes for *Pulmonaria* species: *P. murinii* with 2*n* = 2*x* = 14, 2.31 pg/2C, *P. obscura* 2*n* = 2*x* = 14, 2.69 pg/2C, *P. officinalis* 2*n* = 2*x* = 16, 2.96 pg/2C and *P. mollis* 2*n* = 2*x* = 18, 3.18 pg/2C. Endopolyploidy varies between species and organs, and we have documented 4C–8C in all four organs and up to 32C (64C) endopolyploid nuclei in stems at least in some species. Two species with lower genome sizes tend to have higher endopolyploidy levels than their closest relatives. Endoreplication-generated tissue-specific mean DNA content is increased and more balanced among species in all four organs compared to genome size. Our results argue for the narrow relationship between genome size and endopolyploidy in the present plant group within the genus *Pulmonaria*, and endopolyploidization seems to play a compensatory developmental role in organs of related morphologically similar species.

## Teaser

In plants, a negative correlation between species-specific genome size and endopolyploidy level is assumed. We shed light on this topic, exploring both cytogenetic features in two pairs of morphologically similar diploid–dysploid species of *Pulmonaria*, *P. obscura*–*P. officinalis*and *P. mollis*–*P. murinii*. We have recognized divergent genome sizes and extensive endopolyploidy, and the latter is suggested to be associated with the geophytic growth form of Pulmonarias. Our results provide evidence for the narrow relationship between genome size and endopolyploidy: species with lower genome sizes tend to have greater endopolyploidy levels and vice versa. Endopolyploidization seems to play a compensatory developmental role in organs of related Pulmonarias.

## Introduction

Genome size is a species-specific trait, commonly defined as DNA content in unreplicated gametic holoploid cells (1C value) (Greilhuber *et al.* 2005). An increase in genome size requires a proportionally enlarged nuclear space in cells, and, therefore, it results in so-called nucleotypic effects. These correlate with micro- and macromorphological, physiological and ecological plant variations at cellular, tissue and whole-organism levels (Beaulieu *et al.* 2007, 2008; Knight *et al.* 2010; Šímová and Herben 2012). It was observed that the larger the genome size, the larger the cells (including various microscopic plant structures, such as guard cells and pollen grains), and often also entire plant bodies. In angiosperms, genome size varies from 0.06 pg (1C value) in *Genlisea tuberosa* (Fleischmann *et al.* 2014) to 152.23 pg (1C) in *Paris japonica* (Pellicer *et al.* 2010). Genome size is a stable organismal parameter, heritable from generation to generation and usually with minimal intraspecific variation (if not associated with intraspecific ploidy variation). The ploidy-independent intraspecific genome size variation is believed to be due mainly to the chromosomal gains or losses, sex-determining chromosomal variations and activity of various DNA transposons or retrotransposons (Doležel and Göhde 1995; Ma and Bennetzen 2004; Schmuths *et al.* 2004; Bilinski *et al.* 2018; Varela-Álvarez *et al.* 2021). However, DNA content may vary among functionally diverse cells of plant body, usually in a saltate way, and this phenomenon is known as endopolyploidy.

Endopolyploidy is a general term defined as a result of exponential multiplication of nuclear DNA, by the factor 2^*n*^ (*n*—number of so-called endocycles), without mitosis (D’Amato 1964). It is described as a presence of cells of various ploidy levels within an organism and is generated by several processes, endoreplication, partial endoreplication, endomitosis and cell fusion (D’Amato 1989; El Maâtaoui and Pichot 1999, Joubѐs and Chevalier 2000, Trávníček *et al.* 2015, Brown *et al.* 2017). Endoreplication (also endoreduplication) and endopolyploidization are both terms specifically used for processes leading to endopolyploidy. Endopolyploidy coincides with cell differentiation, and it is essential for normal development and physiology of many plants (Barow 2006). Plants which have undergone endopolyploidization are called polysomatic plants (Wulf 1936; Smulders *et al.* 1994; Mishiba and Mii 2000), but the term ‘endopolyploid plant’ is also widely used (e.g. Barow 2006). Endopolyploidy is widespread among eukaryotes (Nagl 1976; Neiman *et al.* 2017). Within the plant kingdom, endopolyploidy is common among bryophytes and angiosperms (Barow and Meister 2003; Jovtchev *et al.* 2007; Bainard *et al.* 2012; Maluszynska *et al.* 2013; Skaptsov *et al.* 2017; Bainard *et al.* 2020, Paľová *et al.* 2021) and was studied as cytological and physiological phenomena primarily in model organisms (e.g. in *Arabidopsis thaliana*, *Raphanus sativus* or *Solanum lycopersicum*; Galbraith *et al.* 1991; Melaragno *et al.* 1993; Kudo and Kimura 2002; Pirrello *et al.* 2018; Robinson *et al.* 2018). Its common presence and diverse role in plants were revealed relatively recently (Williamson and Hussey 1996; Kondorosi *et al.* 2000; Barow and Meister 2003; Barow 2006; Jovtchev *et al.* 2007; Lee *et al.* 2009; Bainard *et al.* 2011, 2012; Scholes and Paige 2015; Leitch and Dodsworth 2017; Goga *et al.* 2018; Zonneveld 2019; Zedek *et al.* 2020).

The genus *Pulmonaria* (Boraginaceae) is well known for its medicinal effects, horticultural value and complicated evolutionary patterns, but mostly for its characteristic distylous breeding system present in all the species of the genus (Darwin 1877; Olesen 1979; Richards and Mitchell 1990; Hewitt 1994; Champluvier and Jacquemart 1999; Bennett 2003; Meeus *et al.* 2012, 2016; Jacquemyn *et al.* 2018). *Pulmonaria* comprises 18 species, and 8–11 subspecies can be distinguished (Bolliger 1982). The majority of the species occur mainly in shaded woodland habitats (e.g. *P. obscura*, *P. officinalis*), although some prefer dry and sunny habitats (e.g. *P. murinii*) (Hill *et al.* 2004; Janicka and Kasjaniuk 2013).

From an evolutionary perspective, hybridization and polyploidization have played a significant role in infrageneric speciation processes within the genus *Pulmonaria* (Meeus *et al.* 2016). Chromosome counts (2*n*) vary in species and subspecies from 14 to 38, with a total of 10 different cytotypes recognized and a base chromosome number of *n* = *x* = 7 (Sauer 1975; Bolliger 1982). Chromosome counts are used for identification of species in the genus and provide evidence of polyploidization and the presence of dysploid species with incomplete sets of or deviations from the basic chromosome number (aneuploid species in Sauer 1975).

**Figure 1.**
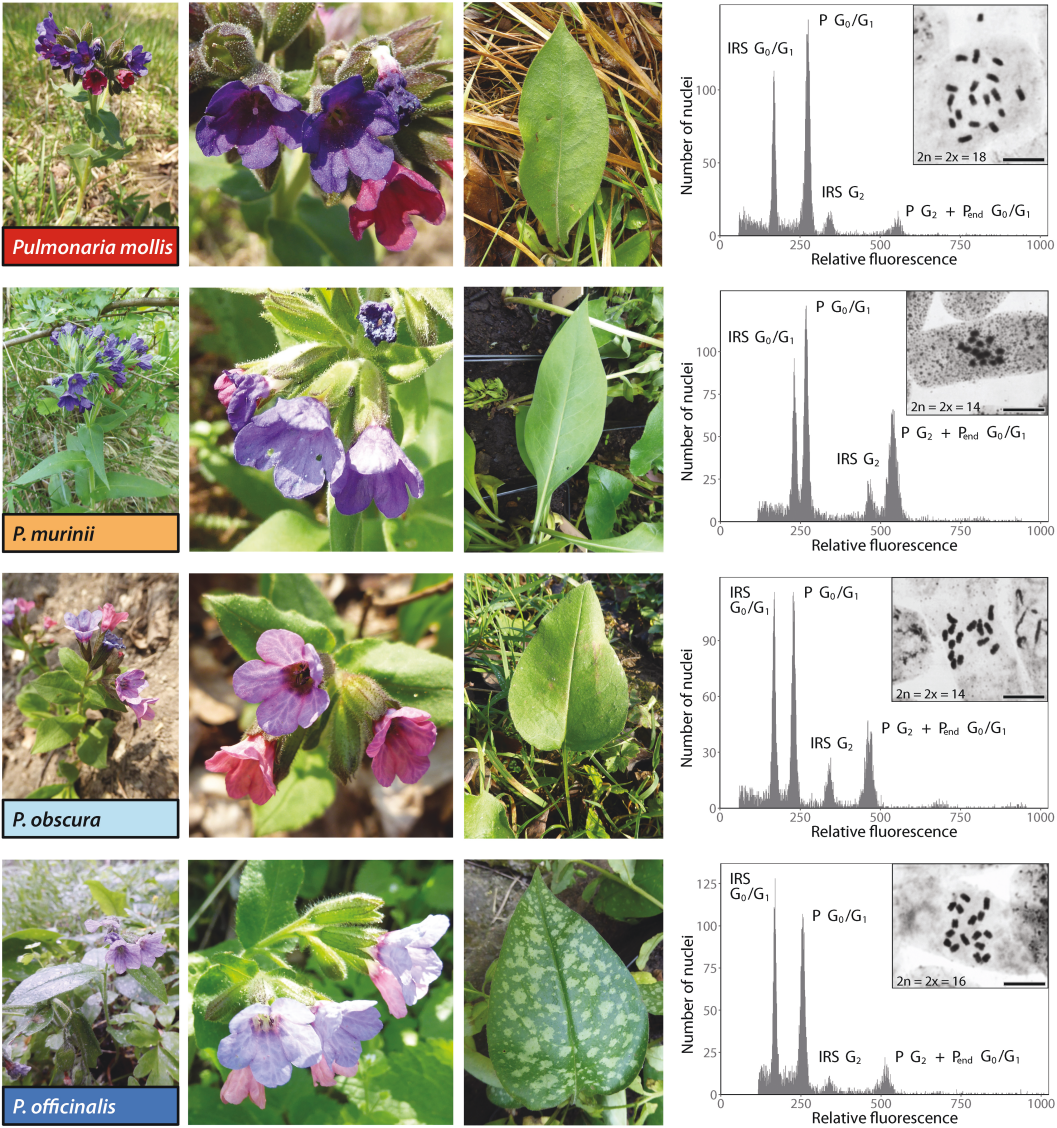
Overview of morphological and cytological variation of four *Pulmonaria* species, *P. mollis* (2*n* = 18), *P. murinii* (2*n* = 14), *P. obscura* (2*n* = 14) and *P. officinalis* (2*n* = 16) as documented with whole habit of plants (first column of figures), inflorescence (second column), leaf shape (third column) and flow cytometry histograms documenting genome size variation with chromosome figures in insets (fourth column). Species relatedness is expressed with different colours, *P. mollis* and *P. murinii* in shades of red, *P. obscura* and *P. officinalis* in shades of blue, while genome size similarity is expressed with different colour intensity, *P. murinii* and *P. obscura* with smaller genomes in lower colour intensity (orange and pale blue, respectively), *P. mollis* and *P. officinalis* with larger genomes with higher colour intensity (red and blue, respectively). G_0_/G_1_, peak of embryo nuclei in G_0_/G_1_ cell cycle phase; G_2_, peak of embryo nuclei in G_2_ cell cycle phase; IRS, internal reference standard; P, *Pulmonaria*.

Five species of *Pulmonaria* are widespread in Central Europe, of which two pairs of related taxa occur more frequently. We can recognize two groups *P. mollis*–*P. murinii* and *P. officinalis*–*P. obscura*, based on morphological inspection of inflorescence, summer leaves (Fig. 1) and trichomes. Indeed, close morphological relationships between taxa within these two pairs are reflected in the frequent merging of each pair of species into one taxon, *P. mollis* and *P. officinalis*. However, karyological data provide the ability to distinguish the four species. *Pulmonaria murinii* was recognized as separate from *P. mollis* based on karyological differences (*P. murinii*, 2*n* = 14, and *P. mollis*, 2*n* = 18) (Májovský and Hegedüšová 1993). Similarly, *P. obscura* differs karyologically from *P. officinalis* (*P. obscura*, 2*n* = 14, and *P. officinalis*, 2*n* = 16). Morphological habit variation within this group of species contrasts with cytogenomic data, chromosome numbers and genome size. Morphological similarity of *Pulmonaria* species reflects the species’ close phylogenetic relationships (Meeus *et al.* 2016); *P. murinii* is more closely related to *P. mollis*, and *P. obscura* is more closely related to *P. officinalis*.

Species of the *Pulmonaria* inhabitating Central Europe are ephemeroidal early-spring plants with features of geophytism. Geophytism seems to be associated with large genome size. Veselý *et al.* (2012) determined the mean 2C DNA content of 21.04 pg for 234 analysed geophytic species, which is more than the mean 2C DNA content of 10.26 pg for 10 770 plant species (The Plant DNA C-Values database; Pellicer and Leitch 2020). However, species of *Pulmonaria*, even with geophytic growth, have very low to low genome sizes (categories according to Leitch *et al.* 1998; Soltis *et al.* 2003) compared to the rest of the angiosperms (range 0.12–304.46 pg/2C; Temsch *et al.* 2021), ranging from 2.57 to 4.27 pg/2C (Kobrlová and Hroneš 2019; Šmarda *et al.* 2019; Zonneveld 2019).

Our knowledge of endopolyploidy variation in various plant groups is quite limited. The recent study of Zonneveld (2019) reports the presence of endopolyploid nuclei on flow cytometry (FCM) records in most of Boraginaceae, including *Pulmonaria* (4C and 8C nuclei in *P. officinalis* and *P. rubra*). These data suggest that endopolyploidy may be a common feature in species of *Pulmonaria*.

In the present study, we analysed two cytologically variable, and morphologically and ecologically contrasting pairs of *Pulmonaria* species, *P. obscura*–*P. officinalis* and *P. mollis*–*P. murinii*, growing in regions of southern Slovakia (Central Europe). We aim (i) to characterize these species cytogenetically, i.e. to determine their genome size and chromosome numbers; (ii) to investigate the degree of endopolyploidy in typical plant organs, including the root, stem, leaf, calyx and corolla; and (iii) to comprehensively characterize and compare the level of endopolyploidy and DNA content in various organs of all four species in relation to species’ evolutionary relationships and genome size variation. The new precise data may help to better understand the relationships of endopolyploidization to plant cytological and morphological forms in related species of *Pulmonaria*. New insights may provide evidence on the evolutionary divergence in *Pulmonaria* and may help to better design morphological comparative investigations in future studies.

## Materials and Methods

### Plant material

In the present study, we have used plant material of four species of *Pulmonaria*, *P. mollis*, *P. murinii*, *P. obscura* and *P. officinalis*. Plants in the mid-flowering stage were used in all investigations. Living plant material was collected in April and May of 2018 for chromosome counts and FCM analyses of endopolyploidy. Each of the four species was represented by four or five populations sampled in various parts of southern range of Slovakia (Central Europe). For each species and locality, five plants were collected. The plants were then potted and cultivated outdoors in the Botanical Garden of Pavol Jozef Šafárik University in Košice. Herbarium specimens of plants used for flow-cytometric analyses are deposited in Herbarium of the Botanical Garden of P. J. Šafárik University, Košice, Slovakia (KO acronym; Thiers 2021+). Three localities were resampled for FCM genome size determination in April of 2020 to ensure that each species was represented by three individuals. Complete data on collections are given in Table 1.

**Table 1.** Plant material summary (*Origin, collection data*) and number of individuals (*Number of plants*) used for chromosome number (*Chromos*), genome size (*GS*) and endopolyploidy (*Endopoly*) determinations.

Species Origin, collection data	Number of plants		
	Chromos	GS	Endopoly
*Pulmonaria mollis*			
Dargov, Biele Studničky, a meadow below the petrol station, 282 m a. s. l., 48°44'07.1"N, 21°34'13.3"E, 7295c, 19 April 2018, MD	—		5
Gemerská Hôrka, a meadow NW of the village, 270 m a. s. l., 48°32'40.3"N, 20°21'51.6"E, 7488c, 22 April 2018, VF	3		5
Košice, Botanical garden, a meadow near the tennis court, 260 m a. s. l., 48°44'12.5"N, 21°14'10.3"E, 7293c, 12 April 2018, VF, LK	4		5
Košice, Botanical garden, west oriented slope above glasshouses, 260 m a. s. l., 48°44'12.5"N, 21°14'10.3"E, 7293c, April 2020, VK	—	3	—
Trnava pri Laborci, a meadow below the castle hill, near the brook, 139 m a. s. l., 48°48'53.2"N, 21°56'34.3"E, 7197d, 19 April 2018, MD	1		5
*Pulmonaria murinii*			
Cejkov, Vlčia hora Mt., a meadow on the top, 243 m a. s. l., 48°28'23.4″N, 21°45'22.9″E, 7596b, 15 April 2018, MD	1		5
Domica, a meadow near the road, 353 m a. s. l., 48°28'35.7"N, 20°28'23.2"E, 7588b, 28 April 2018, VF	1		5
Domica, edge of the meadow NW of the boundary hill Ružový kopec, 357 m a. s. l., 48°28'28.1"N, 20°28'37.1"E, 7588b, 1 April 2020, VF, LK	—	3	—
Leľa, former vineyard, edge of a meadow, 254 m a. s. l., 47°51'12.7"N, 18°45'45.5"E, 8178b, 18 April 2018, VF, LK, MD	2		5
Moldava nad Bodvou, edge of a meadow W of the town, 259 m a. s. l., 48°36'48.6"N, 20°58'42.2"E, 7391d, 3 May 2018, VF, LK	—		5
*Pulmonaria obscura*			
Cejkov, Birec, alder forest near the brook, 175 m a. s. l., 48°27'59.2"N, 21°45'17.5"E, 7596b, 15 April 2018, MD	3		5
Čierna Lehota, forest below Vŕšok Mt., 493 m a. s. l., 48°43'05.8"N, 20°15'49.0"E, 7287d, 15 April 2018, VF, LK	4		5
Dubnica nad Váhom, forest south of Kanada settlement, 454 m a. s. l., 48°56'09.8"N, 18°11'20.2"E, 7075c, 23 April 2018, VF, LK	4		5
Kameňany, forest near the old stone quarry, next to the brook, 258 m a. s. l., 48°35'12.3"N, 20°10'05.0"E, 7487a, 15 April 2018, VF, LK	5		5
Košice, Botanical garden, edge of the forest near the tennis court, 261 m a. s. l., 48°44'13.2"N, 21°14'10.4"E, 7293c, 12 April 2018, VF, LK	3		5
Košice, Botanical garden, west oriented slope above glasshouses, 260 m a. s. l., 48°44'12.5"N, 21°14'10.3"E, 7293c, April 2020, VK	—	3	—
*Pulmonaria officinalis*			
Horné Turovce, little forest near the road north of the village, 146 m a. s. l., 48°08'07.1"N, 18°57'20.8"E, 7879d, 18 April 2018, VF, LK, MD	3		5
Košice, Botanical garden, cultivated individual from a flowerbed, 260 m a. s. l., 48°44'12.5"N, 21°14'10.3"E, 7293c, April 2020, VK	—	1	—
Leľa, near the firehouse, close to the forest, 170 m a. s. l., 47°51'15.8"N, 18°46'05.9"E, 8178b, 18 April 2018, VF, LK, MD	5		5
Nitra, Zobor Mt., forest north from the top, 531 m a. s. l., 48°20'40.3"N, 18°06'13.8"E, 7674d, 26 April 2018, VF, LK	2		5
Nitra, Zobor Mt., forest cca 100 m NW from the sedlo pod Zoborom saddle, near red tourist pathway, 525 m a. s. l., 48°20'42.8"N, 18°06'14.6"E, 7674d, 1 April 2020, VF, LK	—	2	—
Smolenice, forest near the cemetery, 254 m a. s. l., 48°30'40.8"N, 17°25'54.6"E, 7470d, 23 April 2018, VF, LK	2		5

### Karyological analysis

Chromosome numbers were determined in mitotic metaphases of root-tip cells, which were harvested from root-tip meristems from the potted plants (43 in total; Table 1). The root tips were pretreated in a 0.002 M aqueous solution of 8-hydroxyquinoline for 16 h at 4 °C (overnight), then fixed in the mixture of 96 % ethanol and 98 % acetic acid in the ratio 3:1 for 1–24 h. The meristems were washed with distilled water, macerated in 1-N HCl at 60 °C for 6 min and washed again with distilled water for 10 min. Permanent squashes were prepared using the cellophane square method (Murín 1960) and stained in 7 % Giemsa stock solution in Sørensen phosphate buffer for 1 h, washed in distilled water, dried and observed in a drop of immersion oil. A Leica DM 2500 microscope equipped with an HDCE-X5 camera and ScopeImage 9.0 software was used for investigation of chromosomes.

### Flow cytometry, estimation of genome size

The samples for FCM analyses were prepared from a fresh corolla of three *Pulmonaria* plants for each species. Instead of using leaves, which is typical, corollas were used here for genome size analyses because corollas provided better coefficient of variation (CV) results, lower levels of background noise and more symmetrical peaks on FCM histograms. A two-step FCM procedure, consisting of nuclear isolation and staining steps, was used to determine genome size. We selected *S. lycopersicum* ‘Stupické polní tyčkové rané’ (2C = 1.96 pg; Doležel *et al.* 1992) as the internal reference standard (IRS) (Doležel *et al.* 2007; Temsch *et al.* 2021). Approximately 0.75 cm^2^ of a corolla sample and the leaf blade of the IRS were chopped together with a razor blade in a Petri dish in 1 mL of ice-cold general-purpose buffer (GPB): 0.5 mM spermine × 4 HCl, 30 mM sodium citrate, 20 mM MOPS (4-morpholine propane sulfonate), 80 mM KCl, 20 mM NaCl and 0.5 % (v/v) Triton X-100, pH 7.0, prepared according to Loureiro *et al.* (2007). After chopping, the resulting nuclear suspension was filtered through a 42-μm nylon mesh and each sample was stained with propidium iodide (at final concentration 30 μg mL^−1^), a DNA intercalation dye (Doležel and Göhde 1995; Loureiro *et al.* 2007), RNAse (30 μg mL^−1^) and β-mercaptoethanol (2 μL mL^−1^). After ca. 20 min of incubation at 4 °C in darkness, each sample was measured by Partec CyFlow ML (Partec GmbH, Münster, Germany) flow cytometer, equipped with a green solid-state laser operating at 532 nm wavelength and 150 mW, in the Laboratory of Flow cytometry at the Institute of Biological and Ecological Sciences of Pavol Jozef Šafárik University in Košice, Slovakia. FloMax 2.70 software was used for FCM measurements and data analysis. Histograms of the data were displayed on a linear scale (*x*-axis). At least 5000 nuclei per measurement were collected and the CVs of the G_0_/G_1_ peaks of both the samples and the internal standards did not exceed 5 %. The estimation of DNA content of the samples was based on the value of the G_0_/G_1_ peak means of 2C nuclei: DNA amount of the sample = DNA amount of the standard × [(the sample G_0_/G_1_ peak mean)/(the standard G_0_/G_1_ peak mean)] (Doležel and Bartoš 2005). Genome size (2C value) of the species was determined as an average of 2C DNA content of three different plants. 2C DNA content per plant was calculated as an average of the three independent FCM measurements per plant, i.e. each plant was measured three times, on different days (Greilhuber and Obermayer 1997). We detected negligible between-day variation of the three independent FCM measurements (<1.5 %). Genome size data are presented in absolute terms in pg (1C and 2C value) and Mbp (1 pg DNA = 978 Mbp; Doležel *et al.* 2003) and as a relative value referred to the reference standard.

### Flow cytometry, determination of endopolyploidy level

The samples for analyses of endopolyploidy were prepared from different organs, namely roots, stems, leaves, calyces and corollas of mature flowering plants of all four *Pulmonaria* species, for a total of 85 individuals (Table 1). Usually, only one measurement per organ and individual was performed; however, up to five (in one case, even seven) replicates from different lateral roots were examined per individual and then averaged. In total, 530 FCM measurements were performed. For preparation of root, stem and leaf samples, only parts of organs were used. Root sections approximately 1 cm above the root apical meristem and 2–4 cm long (depending on the root thickness) were used. Similarly, stem samples were prepared from about 1–2 cm long section excised from central part of the stem, and leaf samples were prepared from ca. 0.75 cm^2^ of central part of leaf blade of mature rosette leave cut from central part of rosette. For each individual, only one flower was used for sample preparation. The flower was removed from the inflorescence, and floral organs, including the entire corolla and the calyx, were isolated using a razor blade, tweezers and dissecting needles. In the next step, samples of organ pieces for flow-cytometric determination of endopolyploidy were prepared in the same way as samples for genome size estimation, but without adding an IRS. Samples were measured as described above. The histogram data were displayed on a logarithmic scale (*x*-axis). At least 3000 nuclei per measurement were collected.

Evaluation of the extent of endopolyploidy was performed as described in Kolarčik *et al.* (2020) and Fráková *et al.* (2021). The number of nuclei for each ploidy level class (2C, 4C, 8C, etc.) was recorded on FCM histograms, and based on these values, various parameters proposed to evaluate the level of endopolyploidy were calculated (**see****Supporting Information—Note S1** for formula for calculation and meaning of following parameters), mean C value (MCV; Lemontey *et al.* 2000), endoreduplication index (EI; Bainard *et al.* 2012), the mean ploidy level of endopolyploid nuclei (E4P) and proportion of nuclei >2C level (≥4C, given as a percentage). The latter two parameters are modified from those proposed by Dilkes *et al.* (2002) for an analysis of endopolyploidy in seed nutritive tissue, endosperm. The maximal number of endocycles (ECmax) was also recorded. Note that number of endocycles slightly varied, and, therefore, it was lower than ECmax in some cases. The reason may stem from natural origin of plant material, and slight developmental differentiation of plant organs, because precise position of organs was not determined. In addition, we calculated a parameter, called *tissue-specific mean DNA content* (meanDNA), which combines genome size of a species with endopolyploidy level of particular organ. The value of meanDNA is given in picograms (pg) (the formula is proposed in **Supporting Information—Note S1**).

Statistical univariate data analysis was performed to get an overview of the variation in ploidy classes and endopolyploidy parameters (mean and standard deviation are presented). One-way analysis of numerical variance (ANOVA) and Tukey’s HSD pairwise multiple comparison were used to test for differences between means of genome size of different species. Alternatively, non-parametric Kruskal–Wallis test (KW) with the Dunn’s test for pairwise multiple comparison with Benjamini–Hochberg adjustment was used to test for differences between medians of particular endopolyploidy parameters of different species and organs, because data often did not meet assumptions of ANOVA, normality and homoscedasticity. These were controlled by applying the Shapiro–Wilk test and Levene’s test, respectively.

Correlation analysis (Spearman correlation coefficient) was applied to test for relationships among various endopolyploidy parameters. The CV was further calculated as a ratio between standard deviation and mean value for organ-specific mean values (*n* = 4) of each parameter—EI, genome size and meanDNA—to get an overview of variation in those parameters across species under study. Principal component analyses (PCAs) of EI and meanDNA parameters were also calculated to better visualize and compare species and sample variation in both cytological parameters. PCAs were performed on centred (not scaled) data matrices. Data analyses and graphs were produced using packages *gplots* ver. 3.0.1.1 (Warnes *et al.* 2019), *ggplot2* ver. 2.2.1 (Wickham 2009), *PerformanceAnalytics* ver. 1.5.3 (Peterson and Carl 2019), *ade4* (Dray and Dufour 2007) and *vegan* ver. 2.5-4 (Oksanen *et al.* 2019) in R ver. 3.5.3 environment (R Core Team 2019).

## Results

### Chromosome number and genome size determination

The results of the karyological study confirmed known somatic chromosome numbers for four species of *Pulmonaria*. All four species are diploids, *P. mollis* with 2*n* = 2*x* = 18 (altogether determined for eight individuals), *P. murinii* with 2*n* = 2*x* = 14 (four individuals), *P. obscura* with 2*n* = 2*x* = 14 (19 individuals) and *P. officinalis* with 2*n* = 2*x* = 16 (11 individuals) (Fig. 1; Table 1). Only one individual of *P. officinalis* deviates from supposed chromosome count; it was shown to be an aneuploid with 2*n* = 2*x* = 15 chromosomes.

Genome sizes (mean 2C value) of studied species differed significantly (ANOVA, *F*(3, 8) = 689.7, *P* < 0.001) and are reported here in ascending order: *P. murinii* = 2.31 pg, *P. obscura* = 2.69 pg, *P. officinalis* = 2.96 pg and *P. mollis* = 3.18 pg (Table 2; **see****Supporting Information—Table S1**). The variation between the highest and the lowest genome size value spanned 37.7 % (2.31 pg of *P. murinii* represents 100 %). DNA content calculated per chromosome also differed significantly among species (ANOVA, *F*(3, 8) = 135.3, *P* < 0.001). Chromosome size (mean DNA content per chromosome) varies similarly as genome size, but with a different order, *P. murinii* (0.165 pg per chromosome) < *P. mollis* (0.176 pg) < *P. officinalis* (0.185 pg) < *P. obscura* (0.192 pg) (Table 2).

**Table 2.** Genome size of four *Pulmonaria* species. Values for particular parameter followed by different letters are significantly different at *P* = 0.05 (ANOVA and Tukey’s HSD test). 2*n*—species chromosome counts, *N*—number of individuals studied.

Species	2*n*	*N*	Genome size (2C) [pg]		Ratio sample/standard		Genome size (1C) [pg]	Genome size (1C) [Mbp]	DNA content per chromosome [pg]
			Mean ± SD	Min–Max	Mean ± SD	Min–Max	Mean	Mean	Mean
*Pulmonaria mollis*	18	3	3.176 ± 0.006^d^	3.171–3.183	1.620 ± 0.003	1.618–1.624	1.588	1553	0.176^b^
*P. murinii*	14	3	2.311 ± 0.040^a^	2.269–2.347	1.179 ± 0.020	1.158–1.198	1.156	1130	0.165^a^
*P. obscura*	14	3	2.687 ± 0.022^b^	2.673–2.712	1.371 ± 0.011	1.364–1.384	1.343	1314	0.192^d^
*P. officinalis*	16	3	2.963 ± 0.018^c^	2.943–2.978	1.511 ± 0.009	1.501–1.519	1.481	1449	0.185^c^

### Determination of endopolyploidy level and investigation of its variation

**Figure 2.**
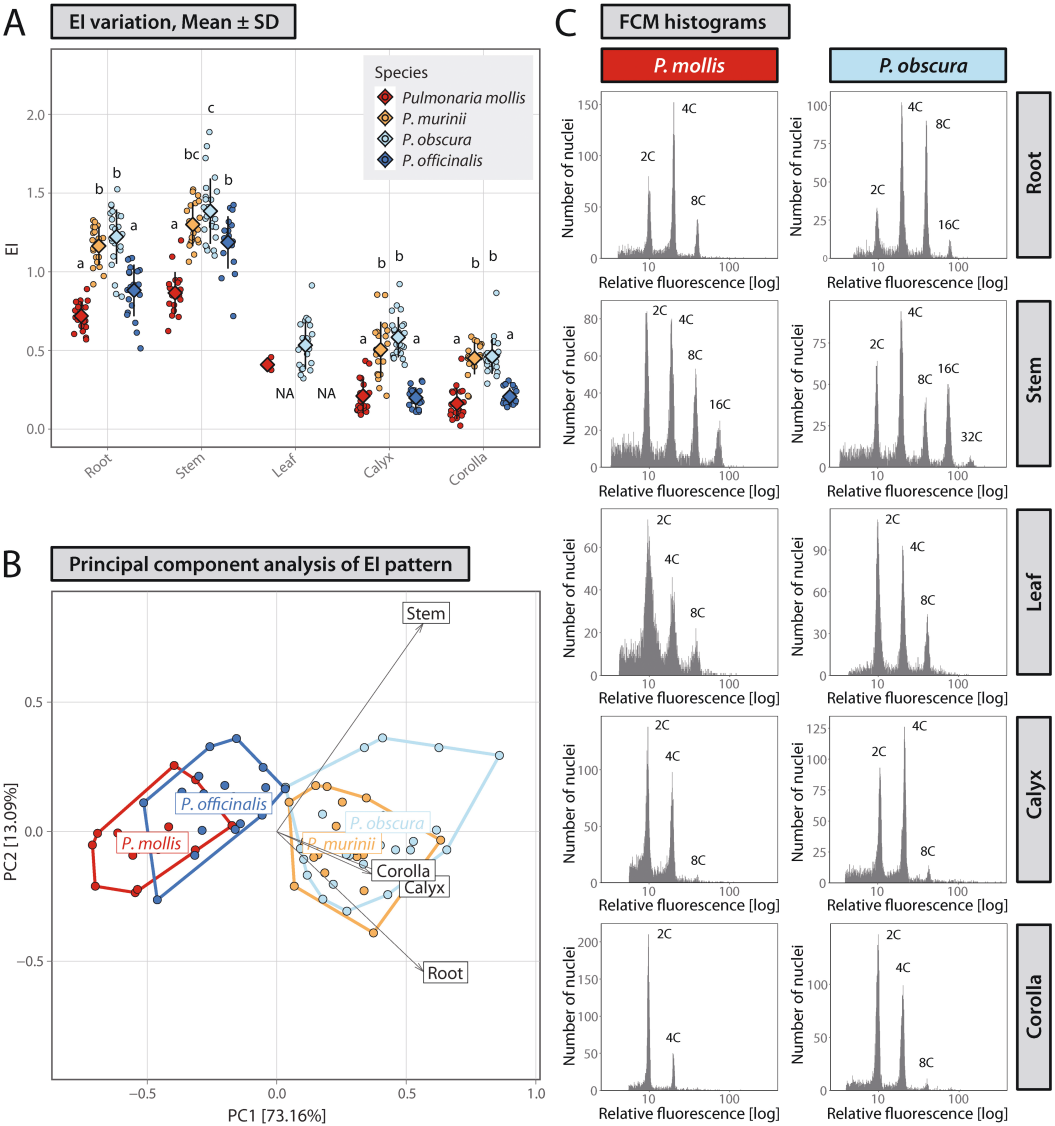
Endopolyploidy level variation in four *Pulmonaria* species, *P. mollis* (2*n* = 18), *P. murinii* (2*n* = 14), *P. obscura* (2*n* = 14) and *P. officinalis* (2*n* = 16). (A) Variation in endoreduplication index, EI, as a measurement of endopolyploidy, calculated values as well as mean ± SD are depicted. Values for particular parameter followed by different letters are significantly different at *P* = 0.05 (Kruskal–Wallis and Dunn’s tests separately performed for each organ). (B) Principal component analysis for EI variation in four organs: root, stem, calyx and corolla. (C) Representative flow cytometry histograms. Species relatedness is expressed with different colours, *P. mollis* and *P. murinii* in shades of red, *P. obscura* and *P. officinalis* in shades of blue, while genome size similarity is expressed with different colour intensity, *P. murinii* and *P. obscura* with smaller genomes in lower colour intensity (orange and pale blue, respectively), *P. mollis* and *P. officinalis* with larger genomes with higher colour intensity (red and blue, respectively).

Flow-cytometric analyses revealed that all four analysed species, *P. mollis*, *P. murinii*, *P. obscura* and *P. officinalis*, are endopolyploid. Endopolyploidy was detected in all analysed organs, and different endopolyploidy patterns were observed (Fig. 2; Tables 3 and 4; **see****Supporting Information—Table S2**). Regarding FCM quality criteria (e.g. low CV for peaks on FCM histograms and low level of background noise) leaves of all four species were problematic in sample preparation and successfully analysed only rarely, probably because of high contents of secondary metabolites, which is known to cause problems in FCM measurements (Noirot *et al.* 2005; Loureiro *et al.* 2006; Bennett *et al.* 2008). Only few measurements were successful for leaves of *P. obscura* and *P. mollis*, and these are presented for comparative purposes (Fig. 2; Tables 3 and 4).

**Table 3.** Endopolyploidy variation of four *Pulmonaria* species and organs investigated in the present study. Number of analysed plants for particular organs and species (*N*) is reported. Proportion (percentage, mean ± standard deviation) is given for nuclei ploidy classes 2C–64C. Data are reported for maximum number of endocycles (ECmax).

Species/organ	*N*	Nuclei ploidy classes						ECmax
		2C [%]	4C [%]	8C [%]	16C [%]	32C [%]	64C [%]	
*Pulmonaria mollis*								
Root	20	35.88 ± 7.00	56.39 ± 6.07	7.73 ± 2.90	—	—	—	2
Stem	19	40.77 ± 6.37	34.65 ± 4.29	21.88 ± 3.12	2.68 ± 3.28	0.02 ± 0.07	—	4
Leaf	3	65.91 ± 2.31	27.25 ± 2.07	6.84 ± 2.41	—	—	—	2
Calyx	20	79.54 ± 10.58	19.88 ± 10.64	0.58 ± 0.87	—	—	—	2
Corolla	20	84.33 ± 8.97	15.07 ± 8.06	0.59 ± 1.20	—	—	—	2
*P. murinii*								
Root	19	15.74 ± 4.15	53.77 ± 5.80	28.91 ± 6.30	1.56 ± 2.08	0.02 ± 0.09	—	4
Stem	20	24.05 ± 4.60	40.55 ± 3.82	19.77 ± 4.00	12.58 ± 3.61	3.02 ± 3.50	0.03 ± 0.12	5
Calyx	20	52.55 ± 16.00	44.50 ± 14.45	2.89 ± 1.87	0.05 ± 0.17	—	—	3
Corolla	20	57.87 ± 9.08	39.25 ± 8.00	2.88 ± 1.47	—	—	—	2
*P. obscura*								
Root	25	18.25 ± 6.26	45.56 ± 7.21	31.91 ± 7.73	4.27 ± 3.74	—	—	3
Stem	25	20.35 ± 4.69	41.46 ± 4.48	20.26 ± 4.89	15.25 ± 6.71	2.65 ± 4.26	0.04 ± 0.20	5
Leaf	22	55.70 ± 8.71	35.37 ± 6.58	8.83 ± 7.48	0.10 ± 0.46	—	—	3
Calyx	25	45.51 ± 12.17	50.74 ± 11.51	3.66 ± 1.10	0.09 ± 0.22	—	—	3
Corolla	25	56.17 ± 10.22	41.61 ± 9.46	2.13 ± 1.00	0.10 ± 0.24	—	—	3
*P. officinalis*								
Root	20	31.95 ± 9.49	48.25 ± 7.26	19.51 ± 8.02	0.29 ± 0.51	—	—	3
Stem	20	29.16 ± 5.20	37.95 ± 3.31	18.99 ± 3.26	12.89 ± 5.07	1.01 ± 1.51	—	4
Calyx	20	80.96 ± 5.98	18.17 ± 5.59	0.87 ± 0.86	—	—	—	2
Corolla	20	80.08 ± 5.12	19.24 ± 4.71	0.69 ± 0.62	—	—	—	2

**Table 4.** Endopolyploidy variation of four *Pulmonaria* species and organs investigated in the present study. Number of analysed plants for particular organs and species (*N*) is reported. Data (mean ± standard deviation) are reported for four endopolyploidy parameters, endoreduplication index (EI), mean C value (MCV), mean ploidy of endopolyploid nuclei (E4P) and estimate of initiation of the endocycle (≥4C), as well as tissue-specific mean DNA content (meanDNA). Values for particular parameter followed by different lower case and upper case letters are significantly different at *P* = 0.05 between species (within the organ) and organs (within the species), respectively (Kruskal–Wallis and Dunn’s tests separately performed for each species and organ). NA—not applied.

Species/organ	*N*	Endopolyploidy parameters				meanDNA [pg]
		EI	MCV	E4P	≥4C [%]	
*Pulmonaria mollis*						
Root	20	0.72 ± 0.09^a/B^	3.59 ± 0.22^a/B^	4.47 ± 0.16^a/B^	64.12 ± 7.00^a/B^	5.70 ± 0.35^a/B^
Stem	19	0.87 ± 0.13^a/B^	4.39 ± 0.54^a/C^	6.00 ± 0.57^a/C^	59.23 ± 6.37^a/B^	6.97 ± 0.85^a/C^
Leaf	3	0.41 ± 0.04^NA/NA^	2.96 ± 0.13^NA/NA^	4.80 ± 0.24^NA/NA^	34.09 ± 2.31^NA/NA^	4.69 ± 0.21^NA/NA^
Calyx	20	0.21 ± 0.11^a/A^	2.43 ± 0.21^a/A^	4.14 ± 0.22^a/A^	20.46 ± 10.58^a/A^	3.86 ± 0.34^b/A^
Corolla	20	0.16 ± 0.10^a/A^	2.34 ± 0.22^a/A^	4.09 ± 0.16^a/A^	15.67 ± 8.97^a/A^	3.71 ± 0.35^b/A^
*P. murinii*						
Root	19	1.16 ± 0.12^b/B^	5.03 ± 0.50^c/B^	5.59 ± 0.49^bc/B^	84.26 ± 4.15^b/C^	5.82 ± 0.58^a/B^
Stem	20	1.30 ± 0.14^bc/B^	6.68 ± 1.04^b/C^	8.13 ± 1.14^b/C^	75.95 ± 4.60^c/B^	7.72 ± 1.20^a/C^
Calyx	20	0.50 ± 0.18^b/A^	3.07 ± 0.39^b/A^	4.24 ± 0.11^ab/A^	47.45 ± 16.00^b/A^	3.55 ± 0.45^a/A^
Corolla	20	0.45 ± 0.10^b/A^	2.96 ± 0.23^b/A^	4.26 ± 0.11^c/A^	42.13 ± 9.08^b/A^	3.42 ± 0.27^a/A^
*P. obscura*						
Root	25	1.22 ± 0.17^b/B^	5.42 ± 0.71^c/B^	6.17 ± 0.73^c/C^	81.75 ± 6.26^b/B^	7.28 ± 0.96^b/B^
Stem	25	1.39 ± 0.21^c/B^	7.00 ± 1.56^b/B^	8.22 ± 1.67^b/D^	79.65 ± 4.69^c/B^	9.40 ± 2.10^b/B^
Leaf	22	0.53 ± 0.15^NA/A^	3.25 ± 0.45^NA/A^	4.76 ± 0.63^NA/B^	44.30 ± 8.71^NA/A^	4.37 ± 0.60^NA/A^
Calyx	25	0.58 ± 0.13^b/A^	3.25 ± 0.27^b/A^	4.29 ± 0.08^b/AB^	54.49 ± 12.17^b/A^	4.36 ± 0.36^c/A^
Corolla	25	0.46 ± 0.11^b/A^	2.97 ± 0.24^b/A^	4.22 ± 0.10^bc/A^	43.83 ± 10.22^b/A^	3.99 ± 0.32^c/A^
*P. officinalis*						
Root	20	0.88 ± 0.16^a/B^	4.18 ± 0.49^b/B^	5.16 ± 0.43^b/B^	68.05 ± 9.49^a/B^	6.18 ± 0.73^a/B^
Stem	20	1.19 ± 0.17^b/C^	6.01 ± 0.92^b/C^	7.61 ± 1.05^b/C^	70.84 ± 5.20^b/B^	8.89 ± 1.36^b/C^
Calyx	20	0.20 ± 0.06^a/A^	2.42 ± 0.14^a/A^	4.17 ± 0.16^ab/A^	19.04 ± 5.98^a/A^	3.58 ± 0.21^a/A^
Corolla	20	0.21 ± 0.06^a/A^	2.43 ± 0.12^a/A^	4.13 ± 0.11^ab/A^	19.92 ± 5.12^a/A^	3.59 ± 0.18^ab/A^

Peaks of 4C and 8C nuclei were detected in all analysed organs of all species (Table 3). It suggests that at least two endocycles occur in endopolyploidisation in species of *Pulmonaria*. The proportion of 16C nuclei was generally lower, but occurred most frequently in stems in all species. 32C nuclei, which correspond to the occurrence of four endocycles, were noted at very low frequencies in stems in all species and in roots of *P. murinii.* Five endocycles, the largest observed, rarely occurred in stems of *P. murinii* and *P. obscura*, and this was confirmed by the detectable presence of 64C nuclei on histograms.

Various endoreplication parameters showed similar patterns of variation across species and organs with few exceptions (see below). Endoreduplication parameters varied for all organs (Fig. 2; Table 4) and their differences between species and organs within the species were statistically significant (*P* < 0.05). In general, the lowest EI were calculated for corollas and calyces, while the highest ones were calculated for stems and roots. Several organs showed similar or even the same, low EI values. For instances, EI of *P. mollis* and *P. officinalis* were very similar in calyces (mean EI of 0.21 and 0.20, respectively), and in corollas (mean EI of 0.16 and 0.21, respectively). On the other side, similarity in high EI values of these organs was observed between *P. murinii* and *P. obscura* for the corolla (mean EI of 0.45 and 0.46, respectively) and calyx (mean EI of 0.50 and 0.58, respectively). Values for calyces and corollas of *P. murinii* and *P. obscura* were more than 2-fold higher compared to *P. mollis* and *P. officinalis*. We observed high EI values (EI > 0.60) in root and stem and their between-species patterns similar to those in the calyx and corolla. Similar patterns were found in other parameters, MCV, E4P and ≥4C. In summary, endoreduplication indices for all organs of *P. murinii* and *P. obscura*, both with 2*n* = 14 and lower genome size (2.31 and 2.69 pg), were significantly higher than EI of *P. mollis* and *P. officinalis*, with 2*n* = 18, 3.18 pg DNA and 2*n* = 16, 2.96 pg DNA, respectively. PCA based on endoreduplication index retrieved 86.25 % of total variance along the first two PC axes and supported the similarity between the pairs of *P. mollis* and *P. officinalis* and of *P. murinii* and *P. obscura* in endopolyploidy level, respectively (Fig. 2B).

Correlation analysis **[see****Supporting Information—Fig. S1****]** revealed narrow relationships among EI, MCV and E4P parameters (Spearman’s *r* ≥ 0.90 in all comparisons). However, parameter ≥4C slightly deviated from this pattern in the upper range (Spearman’s *r* = 0.75 in comparison with E4P). It is reflected in direct comparison of parameter values. For instance, the proportion of ≥4C nuclei was higher in roots compared to stems in three species (*P. officinalis* is an exception), but more nuclei with higher numbers of endocycles (>4C) were present in stems, which resulted into higher EI, MCV or E4P parameters in stems compared to roots.

Comparison of relative variation between species in all four organs revealed a clear trend: the endopolyploidy level decreased with increasing genome size (Fig. 4). However, this relationship is not supported statistically (*P* > 0.05).

**Figure 3.**
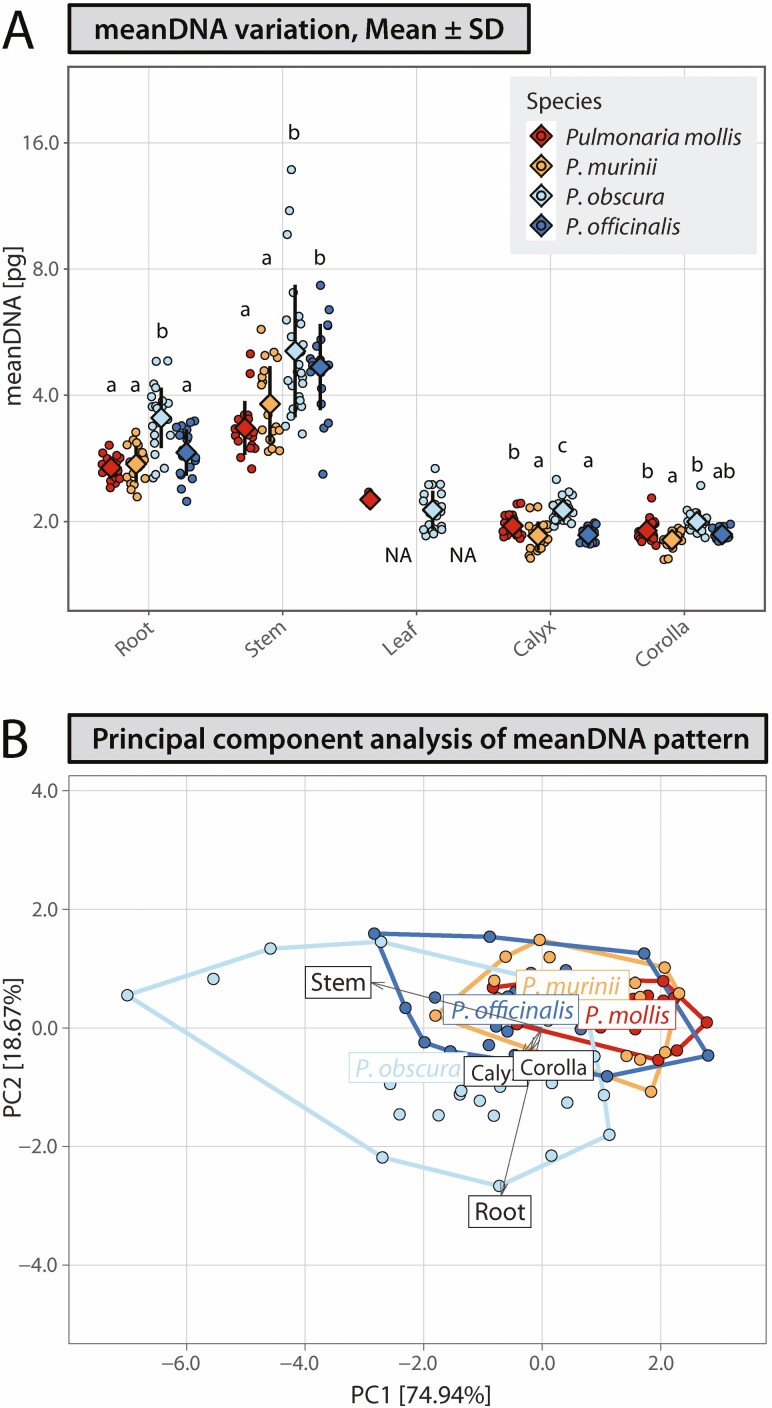
Tissue-specific mean DNA content (meanDNA) variation in four *Pulmonaria* species, *P. mollis* (2*n* = 18), *P. murinii* (2*n* = 14), *P. obscura* (2*n* = 14) and *P. officinalis* (2*n* = 16). (A) Variation in meanDNA, calculated values as well as mean ± SD are depicted. Values for particular parameter followed by different letters are significantly different at *P* = 0.05 (Kruskal–Wallis and Dunn’s tests separately performed for each organ). (B) Principal component analysis for meanDNA variation in four organs: root, stem, calyx and corolla. Species relatedness is expressed with different colours, *P. mollis* and *P. murinii* in shades of red, *P. obscura* and *P. officinalis* in shades of blue, while genome size similarity is expressed with different colour intensity, *P. murinii* and *P. obscura* with smaller genomes in lower colour intensity (orange and pale blue, respectively), *P. mollis* and *P. officinalis* with larger genomes with higher colour intensity (red and blue, respectively).

Tissue-specific mean DNA content varied between organs and species and varied from 3.42 pg on average in corolla of *P. murinii* to 9.40 pg on average in stem of *P. obscura*. Differences in meanDNA between species were statistically significant (*P* < 0.05) in all four tested organs, but the pattern of variation was different compared to EI. While differences in both parameters, EI and meanDNA, are statistically significant, the differences between species were less pronounced with meanDNA than EI parameter (Figs 2 and 3). A high proportion of variation expressed on PC1 vs. PC2 biplots in both PCA analyses of EI (86.25 % of total variance) and meanDNA (93.61 %) allowed for comparison of species similarity patterns in these two parameters across organs. Comparative inspections of PCA grouping patterns showed that, while EI-based PCA grouping showed resemblance of species with lower genome sizes and distinctiveness between species with low and high genome size within the pairs of similar taxa, meanDNA-based PCA ordination demonstrates that the specimens of all four species overlapped, with slight deviation of *P. obscura* samples. More balanced endoreplication-generated tissue-specific DNA content among species for each of the investigated organs compared to genome size was confirmed also by decreasing CV values. While CV in genome size for four *Pulmonaria* species, CV_GS_, was 13.41 %, endopolyploidy varied considerably among species, CV_EI_ ranged from 19.23 % for the stem to 53.03 % in the calyx, but led to less among-species variable tissue-specific mean DNA content with CV_meanDNA_ from 6.56 % in the corolla to 13.40 % in the stem **[see****Supporting Information—Fig. S2****]**.

**Figure 4.**
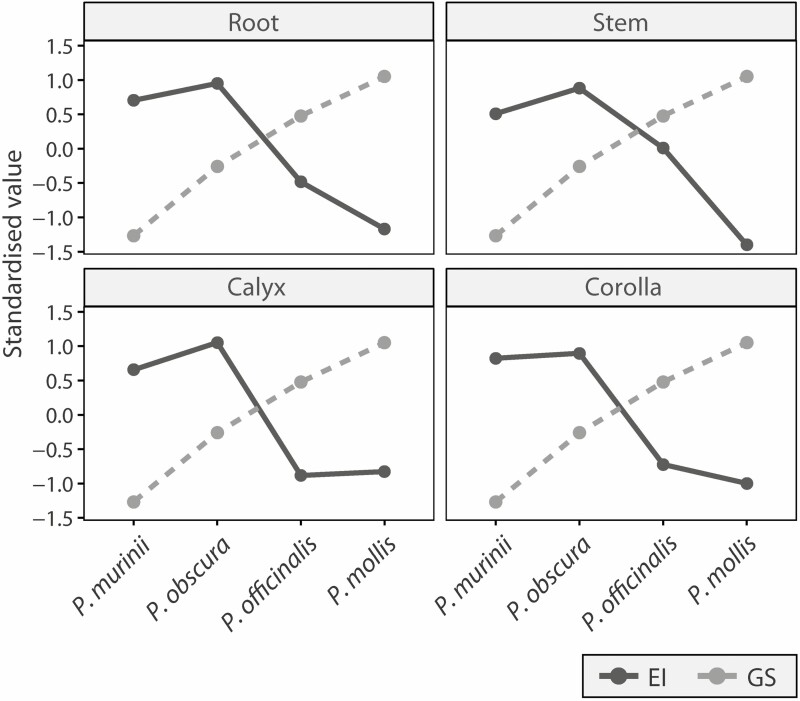
Comparative analysis of genome size and endoreduplication level (expressed as EI) in four species of *Pulmonaria*, *P. mollis* (2*n* = 18), *P. murinii* (2*n* = 14), *P. obscura* (2*n* = 14) and *P. officinalis* (2*n* = 16) and in four investigated organs: root, stem, calyx and corolla. Data were standardized to the zero mean and unit variance prior to plotting.

## Discussion

### Relationship between *Pulmonaria* species and cytogenetic variation

The evolution of the genus *Pulmonaria* is not yet resolved. Since the genus is morphologically quite uniform, karyological data were used widely for species identification. Generally, the basic chromosome number *n* = *x* = 7 is present in most species of the genus (represented with cytotypes 2*n* = 2*x* = 14 and 2*n* = 4*x* = 28) and any others deviating from *x* = 7 are considered dysploid (2*n* = 16, 18, 20, 22, 24, 26, 30, 38) (Sauer 1975; Bolliger 1982). This chromosomal variation may be indicative of hybrid evolution within the genus. Slight morphological differentiation and phylogenetic incongruence in tree topology derived from biparentally inherited nuclear and maternally inherited chloroplast DNA markers suggest that hybridization plays an important role in *Pulmonaria* (Meeus *et al.* 2016; Grünig *et al.* 2021; Liu *et al.* 2022). Indeed, *P. mollis* (2*n* = 18) studied here may be derived from hybrid speciation between two divergent lineages, one including *P. obscura* (2*n* = 14) and other including *P. longifolia* or *P. montana* (2*n* = 22). Future studies incorporating molecular data may thus point to the origin of the chromosome set of 2*n* = 18 (Meeus *et al.* 2016). Nothing is known about the origin of the *P. officinalis* chromosome set 2*n* = 16. While it is divergent from *P. obscura*, with 2*n* = 14, both species are closely related based on molecular data and morphological features (Meeus *et al.* 2013, 2016). Relationships of the fourth species investigated in the present study, *P. murinii* (2*n* = 14), to other three are unknown.

All studied *Pulmonaria* species have small and very small genomes (range 2.31–3.18 pg), according to widely accepted classification proposed by Leitch *et al.* (1998). Genome size data are now available for eight *Pulmonaria* species *P. angustifolia*, *P. longifolia*, *P. mollis*, *P. murinii*, *P. montana*, *P. obscura*, *P. officinalis* and *P. rubra*, and the reports are variable between studies (Table 5). Genome size reports varied for *P. officinalis* (2.90–3.21 pg), *P. obscura* (2.69–3.28 pg) and *P. mollis* (3.18–3.55 pg). Likely, these differences are a result of different methodologies applied, including different standard species used, and partly also because of different plant material (leaves, stems, flowers and different populations) used for genome size investigation. Our new genome size estimates for three species *P. mollis*, *P. obscura* and *P. officinalis* are at lower edge of particular species’ variation but differences in genome size between species fit well to previous reports.

**Table 5.** Genome size reports for *Pulmonaria* (2C value in pg) in various studies.

Species	Zonneveld *et al.* (2005)	Meeus *et al.* (2013)	Pustahija *et al.* (2013)	Zonneveld (2019)	Kobrlová and Hroneš (2019) ^a^	Šmarda *et al.* (2019) ^b^	Present study
*P. angustifolia*	—	—	—	—	3.27	2.57	—
*P. longifolia*	3.80	—	—	—	—	—	—
*P. longifolia* ‘Cebenense’	—	—	—	3.99	—	—	—
*P. mollis*	—	—	—	—	3.55	3.32, 3.43	3.18
*P. montana*	—	—	—	4.27	—	—	—
*P. murinii*	—	—	—	—	—	—	2.31
*P. obscura*	—	—	—	—	2.93	3.28	2.69
*P. officinalis*	3.20	2.90	3.07	3.15	3.12	3.21	2.96
*P. rubra*	—	—	—	2.45	—	—	—

Kobrlová and Hroneš (2019) calculated absolute genome size based on 2C = 2.04 pg for reference standard *Solanum lycopersicum* ‘Stupické polní tyčkové rané’; here given values are recalculated for comparison with our reports with 2C = 1.96 pg (Doležel *et al.* 1992) for the reference standard.

Šmarda *et al.* (2019) calculated absolute genome size based on 2C = 2.077 pg and 2C = 3.159 pg for reference standards *Glycine max* ‘Polanka’ and *Bellis perennis*, respectively; since these values were recalculated against *Oryza sativa* ‘Nipponbare’, here given values are recalculated for comparison with our reports against *Solanum lycopersicum* ‘Stupické polní tyčkové rané’ with 2C = 1.96 pg (Doležel *et al.* 1992).

Contrary to genome size, the morphological similarity (size similarity) of chromosomes may reflect species relatedness (Akbarzadeh *et al.* 2021). Chromosome size better reflects species relationships than genome size in present Pulmonarias. But the cases, where genome size and chromosome size both reflect species relatedness, were also reported (Siljak-Yakovlev *et al.* 2017). Apparently, chromosome rearrangements act differently in both groups of taxa: *P. murinii* (2*n* = 14) has smaller chromosomes (0.165 pg per chromosome) than *P. mollis* (2*n* = 18 and 0.176 pg). The opposite pattern was seen in *P. officinalis* and *P. obscura*. While *P. officinalis* (2*n* = 16) has a higher number of chromosomes than *P. obscura* (2*n* = 14), the chromosomes of the former are smaller than those of the latter (0.185 vs. 0.192 pg per chromosome). This has raised two questions: (i) has dysploidy accompanied genome evolution of *Pulmonaria* repeatedly in different lineages, and (ii) is the hybrid origin involving dysploids (Grünig *et al.* 2021) a common process in reticulate evolutionary history of some species of *Pulmonaria*. Future rigorous phylogenomic studies may provide insight to these questions.

### *Pulmonaria* species are endopolyploid

The analysed organs (i.e. root, stem, calyx and corolla) of four species of *Pulmonaria*, *P. mollis*, *P. murinii*, *P. obscura* and *P. officinalis* were revealed to be endopolyploid (EI > 0.1; Barow and Meister 2003) with EI values ranging from 0.16 (mean value, corolla of *P. mollis*) to 1.39 (stem of *P. obscura*). Extensive endopolyploidy found in *Pulmonaria* is not surprising given that species of the genus are geophytes and members of Boraginaceae, both groups in which endopolyploidy was already reported.

In geophytes, rapid early-spring growth is associated with seasonal development of storage organs, such as rhizomes, bulbs or tubers, and the initiation of growing buds in the end of the vegetative season that are dormant until next year. Veselý *et al.* (2012, 2013) observed that geophytes are generally plants with large genomes and argued that this feature is an important evolutionary trait because it allows for rapid growth of geophytes through nucleotypic effects on morphological, genetic and ecophysiological processes. However, a few geophytes with small genomes exist, and these taxa may be able to utilize the advantages of endopolyploidy-enhanced nucleotypic effects in their growth and development. Indeed, Kolarčik *et al.* (2020) revealed that some geophytes, such as *Corydalis cava* and *C. solida*, have small genomes (2.06 pg and 1.38 pg, respectively), yet high endopolyploidy levels, and both species contain cells with increased nuclear genomes (i.e. up to 32C level of DNA content) in most of their organs, which is comparable to the DNA content of several high genome-sized geophytes. Species of *Pulmonaria* have genomes of similar size to *Corydalis*, and we also recorded endopolyploidy up to 32C (64C), at least in some of organs. Kolarčik *et al.* (2020) recently proposed that endopolyploidy may serve as prerequisite for the evolution of geophytism in species with small genomes.

Endopolyploidy may play multiple roles in geophytes. Several studies have documented that genome multiplying is associated with stress response of plants (Scholes and Paige 2015; Van de Peer *et al.* 2021). One of the stress factors that should be considered is UV-B radiation. It has harmful effects on plant cells, and, therefore, plants have developed a plethora of UV-B protective mechanisms, including protective structures (e.g. trichomes and waxes), enzymatic processes and secondary metabolites (Hollósy 2002). Recent studies suggest that even endopolyploidy may participate in stress response to UV-B radiation (Gegas *et al.* 2014; Zedek *et al.* 2020), but the mechanism currently remains unclear. *Pulmonaria* or *Corydalis* species are exposed to higher doses of UV-B radiation during vegetative growth in the early spring, when trees have not yet fully developed leaves and the shading tree canopy is limited. It is reasonable to consider that endopolyploidy may allow through various ways plants with small genome sizes to evolve geophytic forms. This hypothesis requires further testing across the larger group of geophytes with smaller genome sizes.

The present study represents the most comprehensive investigation of the level and patterns of endopolyploidy on species of *Pulmonaria*, a representative of Boraginaceae. Among other Boraginaceae, endopolyploidy has been noted in vegetative organs of *Cynoglossum officinale* (Bainard *et al.* 2012) and in endosperm of *Echium vulgare* (Biskup and Izmaiłow 2004), and some *Onosma* taxa (Kolarčik *et al.* 2018). Recently, Zonneveld (2019) reported endopolyploidy in 31 out of 40 investigated species, and 13 out of 16 genera of Boraginaceae, including other species of *Pulmonaria* (he reported the presence of nuclei with no more than 8C DNA). He found the presence up to 16C ploidy classes in genera *Cynoglossum*, *Echium*, *Myosotis* and *Symphytum*. Notably, Boraginaceae is related to Solanaceae, which is one of the most economically important groups of polysomatic plants, such as *Capsicum*, *Nicotiana* or *Solanum* (Cheniclet *et al.* 2005; Ogawa *et al.* 2010; Fráková *et al.* 2021). We may expect that endopolyploidy may be common feature in the developmental processes across various genera of Boraginaceae.

### Compensatory role of endopolyploidy in Pulmonarias?

In the present study, similarity in endopolyploidy levels among species of *Pulmonaria* does not reflect species relatedness (i.e. *P. murinii*–*P. mollis* and *P. obscura*–*P. officinalis*), but rather cytogenetic similarity in genome size (i.e. lower genome size in both *P. murinii* and *P. obscura* vs. higher genome size in *P. mollis* and *P. officinalis*). Apparently, these cytogenetic features are ultimately associated, and they negatively correlate in a group of related taxa. This leads to an increase in DNA content in plant tissues, which may be of biological and evolutionary significance (Bainard *et al.* 2012). Hence, we developed here a genomic parameter which combines endopolyploidy level and species-specific genome size into one value—so-called tissue-specific mean DNA content (meanDNA). This parameter may be useful in endopolyploidy research to compare DNA content in specific organs and tissues in comparative investigation of nucleotypic effects in developmental processes, which may be more suitable than comparisons of the effect of basic organismal DNA content—genome size.

Considering variation across species and organs, we observed decreased variation in tissue-specific mean DNA content compared to genome size of species or endoreduplication level (expressed as EI) reached in particular organs (Figs 2 and 3). Notably, lower CV_meanDNA_ was calculated for calyces (9.80 %) and corollas (6.55 %), two organs which at least partly participate in reproduction processes of species of *Pulmonaria* as visual insect attractants and nutlets protection. These values represent a considerable decrease when compared to genome size variation CV_GS_ = 13.41 % and notably endopolyploidy level, CV_EI_ = 49.30 % and CV_EI_ = 53.03 % in corollas and calyces, respectively **[see****Supporting Information—Fig. S2****]**. Similar effects were also observed in roots and stems. This is corroborated in PCA analyses (Figs 2B and 3B). Despite endopolyploidy being highly variable among the four *Pulmonaria* species, similar and higher EI values were recorded in taxa with smaller genome sizes within the two species pairs, *P. murinii*–*P. mollis* and *P. obscura*–*P. officinalis*, which results into lower among-taxa variation in tissue-specific mean DNA. This raises the question: does endopolyploidisation function in tissues as a process, acting to increase cellular DNA content to a level that allows for the optimal utilization of nucleotypic effects in organ development? As a result, we have observed higher and moreover more balanced DNA content among species in particular tissues compared to the basic organismal genome size. The analyses performed in the present study cannot explain the observed lower variation of tissue-specific mean DNA content and assess its significance for biological and evolutionary patterns in the genus. But it is a common part of developmental processes in various organs of species of *Pulmonaria*. All the studied species are morphologically very similar, and the similarity is even pronounced in the case of the perianth (i.e. calyx and corolla), the organs where the differences in tissue-specific mean DNA content is lower when compared to vegetative organs. Therefore, it may be reasonable to suggest that more rounds of endoreplication may work as a compensatory process to lower genome size. Compensatory effects of higher endopolyploidy levels have been suggested for species with low genome size, either in geophytes (otherwise typically with larger genome sizes) or in mosses, which possess small genome sizes. This is even decreased by specific life cycles, which includes vegetative organs with reduced chromosomal complement, gametophytic life stage (Nagl 1976; Paľová *et al.* 2021). Plants may thus benefit from the advantages of higher DNA content in cells created via endopolyploidisation.

## Conclusion

We have shown that endopolyploidy is present across *Pulmonaria* in Central Europe, and it is suggested that it is associated with their geophytic growth form. We have observed that endopolyploidy levels vary between species, and species with lower genome sizes tend to have greater endopolyploidy levels and vice versa.

A notable result of the present study is that various levels of endopolyploidy in *Pulmonaria* are negatively associated with the genome sizes of the investigated species. Indeed, the studied species of *Pulmonaria* with lower genome sizes undergo more endocycles than species with higher genome sizes, regardless of phylogenetic relationships. We have calculated tissue-specific mean DNA content per tissue, which is less variable among taxa for particular organs compared to genome size or endopolyploidy level. Understanding the relationship between tissue-specific mean DNA content and plant morphology requires further research. To this respect, endopolyploidy seems to play some compensatory role to variable basic organismal DNA content—genome size in species of *Pulmonaria*, although limited sampling in the present study does not allow a more general statement across the entire genus to be made. The present study argues for the narrow relationships of genome size and endopolyploidy in related species. Additional studies are necessary to elucidate the role of endopolyploidy-generated DNA content and modifications within and across various organs and tissues and in connection to evolutionary relationships.

## Supporting Information

The following additional information is available in the online version of this article—

**Note S1.** In the study, endopolyploidy level was assessed through various approaches. The number of nuclei for each ploidy level class (2C, 4C, 8C, etc.) was recorded, and four different indices were calculated based on the recorded number of nuclei of each ploidy level class.

**Table S1**. Data of flow cytometry genome size measurements. Species (*Species*), individual Plant and repetitions (*Plant_repetition*) are indicated. Number of nuclei (*Area*), Mean channel value (*Mean*) and coefficient of variation (*CV*, given in %) are reported for FCM peaks of internal reference standard *Solanum lycopersicum* 'Stupické polní tyčkové rané' (*Lyc*) and sample of *Pulmonaria* (*Pulm*).

**Table S2**. Data of flow cytometry endopolyploidy screening. Species (*Species*), origin of plant material (*Locality*, see Table 1 for locality codes), individual plant (*Plant*), organ (*Organ*) and repetitions for an organ (*ORG_rep*) are indicated. Nuclei records for 2C-64C ploidy classes (*2C-64C*) are reported.

**Figure S1**. Correlation analyses of endopolyploidy parameters, endoreduplication index (EI), mean C value (MCV), mean ploidy level of endopolyploid nuclei (E4P) and proportion of cells with > 2C level (≥4C) calculated based on 530 FCM records. Spearman correlation coefficients significant at p < 0.001 are reported.

**Figure S2**. Comparison of variation (expressed as mean coefficient of variation, CV) of endopolyploidy level (expressed as EI), genome size and tissue-specific mean DNA content (meanDNA) in four species of *Pulmonaria*, *P. mollis* (2*n* = 18), *P. murinii* (2*n* = 14), *P. obscura* (2*n* = 14), and *P. officinalis* (2*n* = 16) for four investigated organs, root, stem, calyx and corolla.

plac036_suppl_Supplementary_Material

## Data Availability

Flow cytometry data are attached as **Supporting Information—Tables S1****and****S2**.

## Notes

Evolution & Diversity. Chief Editor: Jeremy Beaulieu
