# Supplementary material for: Genome size and endoreplication in two pairs of cytogenetically contrasting species of Pulmonaria (Boraginaceae) in Central Europe
Source: AoB Plants. 2022 Aug 18;14(5):plac036. doi: 10.1093/aobpla/plac036 (PMC9476981; doi:10.1093/aobpla/plac036)
Supplement: plac036_suppl_Supplementary_Material [file plac036_suppl_supplementary_material.pdf]

SUPPORTING INFORMATION

Authors: Lukáš Koprivý, Viera Fráková, Vladislav Kolarčik, Lenka Mártonfiová, Matej Dudáš and Pavol Mártonfi

Study: Genome size and endoreplication in two pairs of cytogenetically contrasting species of *Pulmonaria* (Boraginaceae) in Central Europe.

Supporting Information Note S1

In the study, endopolyploidy level was assessed through various approaches. The number of nuclei for each ploidy level class (2C, 4C, 8C, etc.) was recorded, and four different indices were calculated based on the recorded number of nuclei of each ploidy level class:

Mean C value (Lemontey et al., 2000) or mean C-level (Jovtchev et al., 2007):

$$\mathrm{MCV} = \frac{[(2 \times \mathrm{n2C}) + (4 \times \mathrm{n4C}) + (8 \times \mathrm{n8C}) + (16 \times \mathrm{n16C}) \ldots]}{(\mathrm{n2C} + \mathrm{n4C} + \mathrm{n8C} + \mathrm{n16C} \ldots)}$$

The endoreduplication index (Bainard et al., 2012) or cycle value (Barow and Meister, 2003):

$$\mathrm{EI} = \frac{[(0 \times \mathrm{n2C}) + (1 \times \mathrm{n4C}) + (2 \times \mathrm{n8C}) + (3 \times \mathrm{n16C}) \ldots]}{(\mathrm{n2C} + \mathrm{n4C} + \mathrm{n8C} + \mathrm{n16C} \ldots)}$$

The parameter E4P (based on E6P parameter proposed by Dilkes et al., 2002) is a mean ploidy level of endopolyploid nuclei:

$$E4P = \frac{[(4 \times n4C) + (8 \times n8C) + (16 \times n16C) \ldots]}{(n4C + n8C + n16C \ldots)}$$

The proportion of cells with > 2C level (given as a percentage, based on %E parameter proposed by Dilkes et al., 2002):

$$\geq 4C = 100 \times \frac{(n4C + n8C + n16C \ldots)}{(n2C + n4C + n8C + n16C \ldots)}$$

Tissue-specific mean DNA content (meanDNA):

$$meanDNA = \frac{[(2 \times GS \times n2C) + (4 \times GS \times n4C) + (8 \times GS \times n8C) \ldots]}{(n2C + n4C + n8C \ldots)}$$

where GS is genome size of respective species given as 1C value, and n2C, n4C, n8C… are nuclei counts of corresponding ploidy level classes (2C, 4C, 8C…).

Supporting Information Table S1

Table S1 Data of flow cytometry genome size measurements. Species (*Species*), individual Plant and repetitions (*Plant_repetition*) are indicated. Number of nuclei (*Area*), Mean channel value (*Mean*) and coefficient of variation (*CV*, given in %) are reported for FCM peaks of internal reference standard *Solanum lycopersicum* 'Stupické polní tyčkové rané' (*Lyc*) and sample of *Pulmonaria* (*Pulm*).

Species	*Plant_repetition*	*Area_Lyc*	*Mean_Lyc*	*CV_Lyc*	*Area_Pulm*	*Mean_Pulm*	*CV_Pulm*
P_mollis	Pmol1_rep1	1370	195.27	4.41	1775	317.87	3.72
P_mollis	Pmol1_rep2	1372	168.94	3.36	2469	274.14	2.83
P_mollis	Pmol1_rep3	1064	197.06	3.64	2042	319.45	3.30
P_mollis	Pmol2_rep1	1926	192.29	3.29	1888	309.84	3.28
P_mollis	Pmol2_rep2	1325	206.98	3.29	3065	334.47	2.94
P_mollis	Pmol2_rep3	2792	181.66	3.70	2257	295.39	3.32
P_mollis	Pmol3_rep1	1707	165.16	3.61	1834	269.37	3.40
P_mollis	Pmol3_rep2	1509	186.18	3.49	1371	299.28	3.54
P_mollis	Pmol3_rep3	1355	184.68	3.90	1672	299.09	3.60
P_mollis	Pmur1_rep1	1547	238.93	3.90	879	284.83	3.20
P_murinii	Pmur1_rep2	1519	196.24	3.70	1345	235.04	3.62
P_murinii	Pmur1_rep3	1351	251.76	3.39	1586	302.87	3.39
P_murinii	Pmur2_rep1	1300	224.37	3.61	1668	266.78	3.51
P_murinii	Pmur2_rep2	1341	242.48	3.28	1583	285.90	3.37
P_murinii	Pmur2_rep3	1600	222.77	3.30	1773	262.77	3.27
P_murinii	Pmur3_rep1	1445	250.67	3.25	2132	289.92	3.90
P_murinii	Pmur3_rep2	1303	232.00	3.14	2071	268.30	2.76
P_murinii	Pmur3_rep3	1700	217.13	4.28	1445	251.77	3.33
P_obscura	Pobscura1_rep1	1342	185.85	5.24	2176	253.40	4.10
P_obscura	Pobscura1_rep2	1415	169.08	3.47	1691	230.29	2.99
P_obscura	Pobscura1_rep3	1357	224.46	3.47	1322	306.50	3.10
P_obscura	Pobscura2_rep1	1492	203.55	4.10	1357	278.13	3.28
P_obscura	Pobscura2_rep2	3074	198.4	3.72	1322	269.22	4.27
P_obscura	Pobscura2_rep3	3546	187.75	3.52	1331	257.46	4.36
P_obscura	Pobscura3_rep1	1364	209.02	3.98	1553	288.26	3.56
P_obscura	Pobscura3_rep2	2941	195.39	3.65	1376	269.74	4.60
P_obscura	Pobscura3_rep3	3888	186.47	4.10	1306	259.51	4.81
P_officinalis	Poffic2_rep1	1324	180.09	4.73	1965	270.95	4.74
P_officinalis	Poffic2_rep2	2363	193.18	4.65	1332	289.89	4.74
P_officinalis	Poffic2_rep3	2362	166.4	3.45	1434	249.44	3.87
P_officinalis	Poffic3_rep1	1674	168.07	4.53	1311	254.71	4.80
P_officinalis	Poffic3_rep2	3610	195.53	4.31	1248	294.14	5.90

Species	*Plant_repetition*	*Area_Lyc*	*Mean_Lyc*	*CV_Lyc*	*Area_Pulm*	*Mean_Pulm*	*CV_Pulm*
P_officinalis	Poffic3_rep3	1505	166.05	3.22	1383	252.63	3.91
P_officinalis	Poffic4_rep1	1388	169.17	3.34	1873	256.65	3.17
P_officinalis	Poffic4_rep2	1459	225.43	5.11	1980	341.15	4.13
P_officinalis	Poffic4_rep3	1526	190.29	3.90	2012	290.69	3.82

Supporting Information Table S2

Table S2 Data of flow cytometry endopolyploidy screening. Species (*Species*), origin of plant material (*Locality*, see Table 1 for locality codes), individual plant (*Plant*), organ (*Organ*) and repetitions for organ (*ORG_rep*) are indicated. Nuclei records for 2C-64C ploidy classes (*2C-64C*) are reported.

Species	*Locality*	*Plant*	*Organ*	*ORG*_rep	*2C*	*4C*	*8C*	*16C*	*32C*	*64C*
P_mollis	MoGH	MoGH1	calyx	calyx	1520	377	32	0	0	0
P_mollis	MoGH	MoGH1	corolla	corolla	2237	418	0	0	0	0
P_mollis	MoGH	MoGH1	root	root_1A	639	1576	341	0	0	0
P_mollis	MoGH	MoGH1	root	root_1B	869	1674	260	0	0	0
P_mollis	MoGH	MoGH1	root	root_1C	441	1674	0	0	0	0
P_mollis	MoGH	MoGH1	root	root_1D	826	1756	37	0	0	0
P_mollis	MoGH	MoGH1	root	root_1E	1039	2223	102	0	0	0
P_mollis	MoGH	MoGH1	stem	stem	1655	1814	969	108	0	0
P_mollis	MoGH	MoGH2	calyx	calyx	1506	712	0	0	0	0
P_mollis	MoGH	MoGH2	corolla	corolla	473	275	38	0	0	0
P_mollis	MoGH	MoGH2	leaf	leaf	1979	894	150	0	0	0
P_mollis	MoGH	MoGH2	root	root_2A	1138	1665	199	0	0	0
P_mollis	MoGH	MoGH2	root	root_2B	1007	1785	215	0	0	0
P_mollis	MoGH	MoGH2	root	root_2C	844	1491	123	0	0	0
P_mollis	MoGH	MoGH2	stem	stem	1799	1895	1062	175	0	0
P_mollis	MoGH	MoGH3	calyx	calyx	1640	282	0	0	0	0
P_mollis	MoGH	MoGH3	corolla	corolla	1844	40	0	0	0	0
P_mollis	MoGH	MoGH3	root	root_3A	957	1796	181	0	0	0
P_mollis	MoGH	MoGH3	root	root_3B	1026	1746	0	0	0	0
P_mollis	MoGH	MoGH3	root	root_3C	764	1670	105	0	0	0
P_mollis	MoGH	MoGH3	root	root_3D	815	1710	114	0	0	0
P_mollis	MoGH	MoGH4	calyx	calyx	1359	364	0	0	0	0
P_mollis	MoGH	MoGH4	corolla	corolla	1530	482	0	0	0	0
P_mollis	MoGH	MoGH4	leaf	leaf	2734	1139	409	0	0	0
P_mollis	MoGH	MoGH4	root	root_4A	943	1606	228	0	0	0
P_mollis	MoGH	MoGH4	root	root_4B	697	1803	268	0	0	0
P_mollis	MoGH	MoGH4	root	root_4C	993	1553	117	0	0	0
P_mollis	MoGH	MoGH4	stem	stem	1506	1416	1033	127	0	0
P_mollis	MoGH	MoGH5	calyx	calyx	878	262	0	0	0	0
P_mollis	MoGH	MoGH5	corolla	corolla	1305	178	0	0	0	0
P_mollis	MoGH	MoGH5	leaf	leaf	2778	1039	244	0	0	0
P_mollis	MoGH	MoGH5	root	root_5A	673	1673	565	0	0	0
P_mollis	MoGH	MoGH5	root	root_5B	824	1809	184	0	0	0
P_mollis	MoGH	MoGH5	root	root_5C	1057	1941	223	0	0	0

Species	*Locality*	*Plant*	*Organ*	*ORG*_rep	*2C*	*4C*	*8C*	*16C*	*32C*	*64C*
P_mollis	MoGH	MoGH5	root	root_5D	896	1739	192	0	0	0
P_mollis	MoGH	MoGH5	root	root_5E	478	1845	129	0	0	0
P_mollis	MoGH	MoGH5	stem	stem	1397	1189	1021	90	0	0
P_mollis	MoKo	MoKo1	calyx	calyx	2023	971	0	0	0	0
P_mollis	MoKo	MoKo1	corolla	corolla	1970	616	0	0	0	0
P_mollis	MoKo	MoKo1	root	root_1A	1518	1153	55	0	0	0
P_mollis	MoKo	MoKo1	root	root_1B	1323	1410	277	0	0	0
P_mollis	MoKo	MoKo1	root	root_1C	1467	1586	239	0	0	0
P_mollis	MoKo	MoKo1	stem	stem	1409	1161	736	0	0	0
P_mollis	MoKo	MoKo2	calyx	calyx	1779	1359	0	0	0	0
P_mollis	MoKo	MoKo2	corolla	corolla	2080	430	0	0	0	0
P_mollis	MoKo	MoKo2	root	root_2A	1111	1587	196	0	0	0
P_mollis	MoKo	MoKo2	root	root_2B	3514	2774	354	0	0	0
P_mollis	MoKo	MoKo2	stem	stem	1145	1657	888	0	0	0
P_mollis	MoKo	MoKo3	calyx	calyx	2108	1023	0	0	0	0
P_mollis	MoKo	MoKo3	corolla	corolla	2145	604	29	0	0	0
P_mollis	MoKo	MoKo3	root	root_3A	1365	1472	273	0	0	0
P_mollis	MoKo	MoKo3	stem	stem	1294	1337	660	61	0	0
P_mollis	MoKo	MoKo4	calyx	calyx	1564	934	67	0	0	0
P_mollis	MoKo	MoKo4	corolla	corolla	2051	645	59	0	0	0
P_mollis	MoKo	MoKo4	root	root_4A	943	1599	444	0	0	0
P_mollis	MoKo	MoKo4	root	root_4B	742	1533	313	0	0	0
P_mollis	MoKo	MoKo4	root	root_4C	739	1550	547	0	0	0
P_mollis	MoKo	MoKo4	root	root_4D	865	1573	243	0	0	0
P_mollis	MoKo	MoKo4	root	root_4E	949	1443	201	0	0	0
P_mollis	MoKo	MoKo4	stem	stem	1300	1363	843	417	0	0
P_mollis	MoKo	MoKo5	calyx	calyx	1942	764	0	0	0	0
P_mollis	MoKo	MoKo5	corolla	corolla	2113	570	45	0	0	0
P_mollis	MoKo	MoKo5	root	root_5A	1269	1675	483	0	0	0
P_mollis	MoKo	MoKo5	root	root_5B	1236	1478	341	0	0	0
P_mollis	MoKo	MoKo5	root	root_5C	1162	1573	264	0	0	0
P_mollis	MoKo	MoKo5	root	root_5D	1508	1338	125	0	0	0
P_mollis	MoKo	MoKo5	root	root_5E	1050	1617	241	0	0	0
P_mollis	MoKo	MoKo5	stem	stem	1806	1725	1138	0	0	0
P_mollis	MoSv	MoSv1	calyx	calyx	4066	383	55	0	0	0
P_mollis	MoSv	MoSv1	corolla	corolla	2082	197	19	0	0	0
P_mollis	MoSv	MoSv1	root	root_1A	1607	1983	91	0	0	0
P_mollis	MoSv	MoSv1	root	root_1B	1104	2008	188	0	0	0
P_mollis	MoSv	MoSv1	stem	stem	1459	1327	788	56	0	0
P_mollis	MoSv	MoSv2	calyx	calyx	1620	222	0	0	0	0
P_mollis	MoSv	MoSv2	corolla	corolla	4910	577	0	0	0	0
P_mollis	MoSv	MoSv2	root	root	1152	1694	216	0	0	0
P_mollis	MoSv	MoSv2	stem	stem	2373	1890	1258	108	18	0
P_mollis	MoSv	MoSv3	calyx	calyx	1624	275	35	0	0	0

Species	*Locality*	*Plant*	*Organ*	*ORG*_rep	*2C*	*4C*	*8C*	*16C*	*32C*	*64C*
P_mollis	MoSv	MoSv3	corolla	corolla	2296	279	0	0	0	0
P_mollis	MoSv	MoSv3	root	root	885	1730	238	0	0	0
P_mollis	MoSv	MoSv3	stem	stem	1533	1755	1311	631	0	0
P_mollis	MoSv	MoSv4	calyx	calyx	2069	244	47	0	0	0
P_mollis	MoSv	MoSv4	corolla	corolla	2823	211	0	0	0	0
P_mollis	MoSv	MoSv4	root	root	1665	1890	95	0	0	0
P_mollis	MoSv	MoSv4	stem	stem	1416	1081	615	64	0	0
P_mollis	MoSv	MoSv5	calyx	calyx	1583	221	0	0	0	0
P_mollis	MoSv	MoSv5	corolla	corolla	2243	210	0	0	0	0
P_mollis	MoSv	MoSv5	root	root	801	1630	247	0	0	0
P_mollis	MoSv	MoSv5	stem	stem	1561	1012	881	136	0	0
P_mollis	MoVi	MoVi1	calyx	calyx	1669	198	0	0	0	0
P_mollis	MoVi	MoVi1	corolla	corolla	1364	179	0	0	0	0
P_mollis	MoVi	MoVi1	root	root	1025	1628	352	0	0	0
P_mollis	MoVi	MoVi1	stem	stem	1488	936	757	77	0	0
P_mollis	MoVi	MoVi2	calyx	calyx	2936	371	34	0	0	0
P_mollis	MoVi	MoVi2	corolla	corolla	2286	231	0	0	0	0
P_mollis	MoVi	MoVi2	root	root_2A	809	1607	249	0	0	0
P_mollis	MoVi	MoVi2	root	root_2B	853	1831	299	0	0	0
P_mollis	MoVi	MoVi2	root	root_2C	485	1768	581	0	0	0
P_mollis	MoVi	MoVi2	root	root_2D	232	1773	316	0	0	0
P_mollis	MoVi	MoVi2	root	root_2E	618	976	212	0	0	0
P_mollis	MoVi	MoVi2	stem	stem	1566	840	603	54	0	0
P_mollis	MoVi	MoVi3	calyx	calyx	1735	174	0	0	0	0
P_mollis	MoVi	MoVi3	corolla	corolla	2253	141	0	0	0	0
P_mollis	MoVi	MoVi3	root	root_3A	1460	1875	347	0	0	0
P_mollis	MoVi	MoVi3	root	root_3B	1257	845	0	0	0	0
P_mollis	MoVi	MoVi3	root	root_3C	994	1705	291	0	0	0
P_mollis	MoVi	MoVi3	stem	stem	1641	1125	709	32	0	0
P_mollis	MoVi	MoVi4	calyx	calyx	1275	180	19	0	0	0
P_mollis	MoVi	MoVi4	corolla	corolla	2396	627	42	0	0	0
P_mollis	MoVi	MoVi4	root	root	856	1672	312	0	0	0
P_mollis	MoVi	MoVi4	stem	stem	1536	1117	602	0	0	0
P_mollis	MoVi	MoVi5	calyx	calyx	1825	288	0	0	0	0
P_mollis	MoVi	MoVi5	corolla	corolla	1577	237	0	0	0	0
P_mollis	MoVi	MoVi5	root	root_5A	836	1727	241	0	0	0
P_mollis	MoVi	MoVi5	root	root_5B	1495	1614	138	0	0	0
P_mollis	MoVi	MoVi5	stem	stem	1577	996	406	20	0	0
P_murinii	MuCe	MuCe1	calyx	calyx	1112	1505	81	0	0	0
P_murinii	MuCe	MuCe1	corolla	corolla	2070	1564	93	0	0	0
P_murinii	MuCe	MuCe1	root	root_1A	555	1950	805	0	0	0
P_murinii	MuCe	MuCe1	root	root_1C	663	1573	576	0	0	0
P_murinii	MuCe	MuCe1	stem	stem	1404	1449	813	662	19	0
P_murinii	MuCe	MuCe2	calyx	calyx	1450	1303	131	0	0	0

Species	*Locality*	*Plant*	*Organ*	*ORG*_rep	*2C*	*4C*	*8C*	*16C*	*32C*	*64C*
P_murinii	MuCe	MuCe2	corolla	corolla	2017	2116	220	0	0	0
P_murinii	MuCe	MuCe2	root	root_2A	561	1553	364	0	0	0
P_murinii	MuCe	MuCe2	stem	stem	953	1693	805	437	0	0
P_murinii	MuCe	MuCe3	calyx	calyx	2397	2715	224	0	0	0
P_murinii	MuCe	MuCe3	corolla	corolla	3433	3006	320	0	0	0
P_murinii	MuCe	MuCe3	root	root_3A	538	1940	1242	223	0	0
P_murinii	MuCe	MuCe3	root	root_3B	416	1445	923	95	0	0
P_murinii	MuCe	MuCe3	root	root_3C	384	1531	1121	124	0	0
P_murinii	MuCe	MuCe3	root	root_3D	342	1911	1590	194	0	0
P_murinii	MuCe	MuCe3	root	root_3E	402	1788	1138	0	0	0
P_murinii	MuCe	MuCe3	stem	stem	823	1477	761	373	0	0
P_murinii	MuCe	MuCe4	calyx	calyx	1645	1989	143	16	0	0
P_murinii	MuCe	MuCe4	corolla	corolla	2046	1317	112	0	0	0
P_murinii	MuCe	MuCe4	root	root_4A	389	1638	664	0	0	0
P_murinii	MuCe	MuCe4	root	root_4C	485	1455	518	0	0	0
P_murinii	MuCe	MuCe4	root	root_4D	373	1665	586	0	0	0
P_murinii	MuCe	MuCe4	root	root_4E	508	1520	332	0	0	0
P_murinii	MuCe	MuCe4	stem	stem	1161	1531	1487	203	0	0
P_murinii	MuCe	MuCe5	calyx	calyx	1499	1743	120	0	0	0
P_murinii	MuCe	MuCe5	corolla	corolla	2215	1329	125	0	0	0
P_murinii	MuCe	MuCe5	root	root	495	1628	1025	80	0	0
P_murinii	MuCe	MuCe5	stem	stem	679	1555	780	441	275	0
P_murinii	MuDo	MuDo1	calyx	calyx	805	701	40	0	0	0
P_murinii	MuDo	MuDo1	corolla	corolla	1585	1371	145	0	0	0
P_murinii	MuDo	MuDo1	root	root_R10	224	1593	891	0	0	0
P_murinii	MuDo	MuDo1	stem	stem	834	1365	828	580	0	0
P_murinii	MuDo	MuDo2	calyx	calyx	1241	598	26	0	0	0
P_murinii	MuDo	MuDo2	corolla	corolla	1008	852	84	0	0	0
P_murinii	MuDo	MuDo2	root	root	572	2065	1379	0	0	0
P_murinii	MuDo	MuDo2	stem	stem	554	1558	502	608	210	0
P_murinii	MuDo	MuDo3_R3	calyx	calyx	1461	512	0	0	0	0
P_murinii	MuDo	MuDo3_R3	corolla	corolla	1639	1130	76	0	0	0
P_murinii	MuDo	MuDo3_R3	root	root_R3A	495	2249	1355	102	0	0
P_murinii	MuDo	MuDo3_R3	stem	stem	882	1599	608	674	146	0
P_murinii	MuDo	MuDo4	calyx	calyx	1424	996	98	0	0	0
P_murinii	MuDo	MuDo4	corolla	corolla	1912	1090	97	0	0	0
P_murinii	MuDo	MuDo4	root	root_R4A	471	1528	794	0	0	0
P_murinii	MuDo	MuDo4	root	root_R4B	328	1641	476	0	0	0
P_murinii	MuDo	MuDo4	root	root_R4C	726	1799	862	0	0	0
P_murinii	MuDo	MuDo4	root	root_R4D	322	1785	881	0	0	0
P_murinii	MuDo	MuDo4	root	root_R4E	215	948	297	0	0	0
P_murinii	MuDo	MuDo4	stem	stem	678	1650	649	323	294	19
P_murinii	MuDo	MuDo5	calyx	calyx	1700	729	26	0	0	0
P_murinii	MuDo	MuDo5	corolla	corolla	2107	2199	162	0	0	0

Species	*Locality*	*Plant*	*Organ*	*ORG*_rep	*2C*	*4C*	*8C*	*16C*	*32C*	*64C*
P_murinii	MuDo	MuDo5	stem	stem	755	1864	699	904	56	0
P_murinii	MuLe	MuLe1	calyx	calyx	1602	2073	124	25	0	0
P_murinii	MuLe	MuLe1	corolla	corolla	1994	1878	198	0	0	0
P_murinii	MuLe	MuLe1	root	root	692	1753	1087	45	0	0
P_murinii	MuLe	MuLe1	stem	stem	1406	2196	993	591	229	0
P_murinii	MuLe	MuLe2	calyx	calyx	494	1684	150	0	0	0
P_murinii	MuLe	MuLe2	corolla	corolla	1948	1474	70	0	0	0
P_murinii	MuLe	MuLe2	root	root_2A	193	1013	1102	363	21	0
P_murinii	MuLe	MuLe2	root	root_2B	562	1646	626	0	0	0
P_murinii	MuLe	MuLe2	stem	stem	1678	1984	875	528	23	0
P_murinii	MuLe	MuLe3	calyx	calyx	979	455	26	0	0	0
P_murinii	MuLe	MuLe3	corolla	corolla	1996	520	0	0	0	0
P_murinii	MuLe	MuLe3	root	root_3A	614	1593	769	52	0	0
P_murinii	MuLe	MuLe3	root	root_3B	422	1571	548	0	0	0
P_murinii	MuLe	MuLe3	root	root_3C	521	1770	614	0	0	0
P_murinii	MuLe	MuLe3	root	root_3D	302	705	334	0	0	0
P_murinii	MuLe	MuLe3	root	root_3E	373	1458	564	41	0	0
P_murinii	MuLe	MuLe3	stem	stem	1657	2393	1223	635	448	0
P_murinii	MuLe	MuLe4	calyx	calyx	1319	893	37	0	0	0
P_murinii	MuLe	MuLe4	corolla	corolla	4892	1204	46	0	0	0
P_murinii	MuLe	MuLe4	root	root_4A	227	1561	765	0	0	0
P_murinii	MuLe	MuLe4	stem	stem	727	1806	746	395	33	0
P_murinii	MuLe	MuLe5	calyx	calyx	1454	936	57	0	0	0
P_murinii	MuLe	MuLe5	corolla	corolla	3233	1919	82	0	0	0
P_murinii	MuLe	MuLe5	root	root	612	1613	537	0	0	0
P_murinii	MuLe	MuLe5	stem	stem	1060	1957	978	635	296	0
P_murinii	MuMB	MuMB1	calyx	calyx	2351	1058	67	0	0	0
P_murinii	MuMB	MuMB1	corolla	corolla	2222	1523	118	0	0	0
P_murinii	MuMB	MuMB1	root	root_1A	956	2483	1955	314	0	0
P_murinii	MuMB	MuMB1	stem	stem	1105	1550	821	520	21	0
P_murinii	MuMB	MuMB2	calyx	calyx	3952	897	71	0	0	0
P_murinii	MuMB	MuMB2	corolla	corolla	2189	1669	54	0	0	0
P_murinii	MuMB	MuMB2	root	root_2A	299	1618	1013	0	0	0
P_murinii	MuMB	MuMB2	root	root_2B	567	1500	1166	267	0	0
P_murinii	MuMB	MuMB2	stem	stem	1304	1984	781	512	0	0
P_murinii	MuMB	MuMB3	calyx	calyx	689	2167	238	0	0	0
P_murinii	MuMB	MuMB3	corolla	corolla	2302	1829	151	0	0	0
P_murinii	MuMB	MuMB3	root	root_3A	371	1527	1601	83	0	0
P_murinii	MuMB	MuMB3	root	root_3B	573	1501	1183	90	0	0
P_murinii	MuMB	MuMB3	root	root_3C	296	1504	1083	85	0	0
P_murinii	MuMB	MuMB3	stem	stem	817	1368	643	395	411	0
P_murinii	MuMB	MuMB4	calyx	calyx	964	1629	58	0	0	0
P_murinii	MuMB	MuMB4	corolla	corolla	2150	1687	38	0	0	0
P_murinii	MuMB	MuMB4	root	root_4B	518	747	1657	72	0	0

Species	*Locality*	*Plant*	*Organ*	*ORG*_rep	*2C*	*4C*	*8C*	*16C*	*32C*	*64C*
P_murinii	MuMB	MuMB4	root	root_4C	662	1880	335	0	0	0
P_murinii	MuMB	MuMB4	stem	stem	2377	3550	1862	1226	258	0
P_murinii	MuMB	MuMB5	calyx	calyx	1639	1398	34	0	0	0
P_murinii	MuMB	MuMB5	corolla	corolla	3480	1545	110	0	0	0
P_murinii	MuMB	MuMB5	root	root_5A	571	1412	833	0	0	0
P_murinii	MuMB	MuMB4	root	root_5B	343	1668	1288	0	0	0
P_murinii	MuMB	MuMB4	root	root_5C	363	1693	335	0	0	0
P_murinii	MuMB	MuMB5	stem	stem	1024	1422	814	517	0	0
P_obscura	ObBZ	ObBZ1	calyx	calyx	1232	918	79	0	0	0
P_obscura	ObBZ	ObBZ1	corolla	corolla	2096	1317	41	0	0	0
P_obscura	ObBZ	ObBZ1	root	root_1A	365	1491	1458	221	0	0
P_obscura	ObBZ	ObBZ1	root	root_1B	473	1551	1588	366	0	0
P_obscura	ObBZ	ObBZ1	root	root_1C	552	2057	1500	337	0	0
P_obscura	ObBZ	ObBZ1	stem	stem	801	1574	702	102	112	0
P_obscura	ObBZ	ObBZ2	calyx	calyx	1317	1254	90	0	0	0
P_obscura	ObBZ	ObBZ2	corolla	corolla	2206	1549	39	0	0	0
P_obscura	ObBZ	ObBZ2	leaf	leaf	2188	1632	681	0	0	0
P_obscura	ObBZ	ObBZ2	root	root_2A	588	1226	510	23	0	0
P_obscura	ObBZ	ObBZ2	root	root_2B	329	1596	1602	111	0	0
P_obscura	ObBZ	ObBZ2	root	root_2C	647	-61	892	193	0	0
P_obscura	ObBZ	ObBZ2	root	root_2D	475	1667	744	38	0	0
P_obscura	ObBZ	ObBZ2	stem	stem	1066	1683	694	721	46	0
P_obscura	ObBZ	ObBZ3	calyx	calyx	983	1627	81	0	0	0
P_obscura	ObBZ	ObBZ3	corolla	corolla	2100	1454	75	0	0	0
P_obscura	ObBZ	ObBZ3	leaf	leaf	1889	1393	576	0	0	0
P_obscura	ObBZ	ObBZ3	root	root_3A	550	776	822	93	0	0
P_obscura	ObBZ	ObBZ3	root	root_3B	410	1312	1277	444	0	0
P_obscura	ObBZ	ObBZ3	stem	stem	826	1510	600	901	99	0
P_obscura	ObBZ	ObBZ4	calyx	calyx	1299	1651	119	0	0	0
P_obscura	ObBZ	ObBZ4	corolla	corolla	2706	1636	50	0	0	0
P_obscura	ObBZ	ObBZ4	root	root	857	1343	1286	669	0	0
P_obscura	ObBZ	ObBZ4	stem	stem	830	1647	671	713	199	0
P_obscura	ObBZ	ObBZ5	calyx	calyx	1096	1366	117	0	0	0
P_obscura	ObBZ	ObBZ5	corolla	corolla	2131	1468	80	13	0	0
P_obscura	ObBZ	ObBZ5	leaf	leaf	2070	1458	370	0	0	0
P_obscura	ObBZ	ObBZ5	root	root_5A	377	1343	1047	127	0	0
P_obscura	ObBZ	ObBZ5	root	root_5B	286	1663	1633	113	0	0
P_obscura	ObBZ	ObBZ5	root	root_5C	447	1720	1128	84	0	0
P_obscura	ObBZ	ObBZ5	stem	stem	715	1540	591	785	17	0
P_obscura	ObCe	ObCe1	calyx	calyx	1386	1315	63	0	0	0
P_obscura	ObCe	ObCe1	corolla	corolla	2254	1335	83	0	0	0
P_obscura	ObCe	ObCe1	leaf	leaf	1431	835	72	0	0	0
P_obscura	ObCe	ObCe1	root	root_1A	166	1457	575	25	0	0
P_obscura	ObCe	ObCe1	root	root_1B	338	645	198	0	0	0

Species	*Locality*	*Plant*	*Organ*	*ORG*_rep	*2C*	*4C*	*8C*	*16C*	*32C*	*64C*
P_obscura	ObCe	ObCe1	stem	stem	1058	2787	1285	1200	852	0
P_obscura	ObCe	ObCe2	calyx	calyx	783	1884	118	0	0	0
P_obscura	ObCe	ObCe2	corolla	corolla	2035	1377	60	0	0	0
P_obscura	ObCe	ObCe2	leaf	leaf	1797	1172	533	77	0	0
P_obscura	ObCe	ObCe2	root	root_2A	344	1563	538	27	0	0
P_obscura	ObCe	ObCe2	root	root_2B	329	1404	749	70	0	0
P_obscura	ObCe	ObCe2	root	root_2C	433	786	466	191	0	0
P_obscura	ObCe	ObCe2	root	root_2D	291	1677	446	0	0	0
P_obscura	ObCe	ObCe2	root	root_2E	1116	2196	1268	106	0	0
P_obscura	ObCe	ObCe2	stem	stem	589	1501	679	800	525	0
P_obscura	ObCe	ObCe3	calyx	calyx	479	2639	215	0	0	0
P_obscura	ObCe	ObCe3	corolla	corolla	1795	2137	130	0	0	0
P_obscura	ObCe	ObCe3	leaf	leaf	1422	714	613	0	0	0
P_obscura	ObCe	ObCe3	root	root_3A	662	1355	989	133	0	0
P_obscura	ObCe	ObCe3	root	root_3B	747	1206	1146	234	0	0
P_obscura	ObCe	ObCe3	root	root_3C	735	1412	990	109	0	0
P_obscura	ObCe	ObCe3	root	root_3D	453	1796	934	104	0	0
P_obscura	ObCe	ObCe3	root	root_3E	274	2019	1321	40	0	0
P_obscura	ObCe	ObCe3	stem	stem	613	1666	1038	734	735	48
P_obscura	ObCe	ObCe4	calyx	calyx	1540	1377	114	0	0	0
P_obscura	ObCe	ObCe4	corolla	corolla	2015	1145	32	0	0	0
P_obscura	ObCe	ObCe4	leaf	leaf	1032	1812	722	0	0	0
P_obscura	ObCe	ObCe4	root	root_4A	93	1207	1108	79	0	0
P_obscura	ObCe	ObCe4	root	root_4C	204	822	474	46	0	0
P_obscura	ObCe	ObCe4	stem	stem	612	1721	588	661	65	0
P_obscura	ObCe	ObCe5	calyx	calyx	1672	981	64	0	0	0
P_obscura	ObCe	ObCe5	corolla	corolla	2384	880	36	0	0	0
P_obscura	ObCe	ObCe5	root	root_5A	657	1443	1054	183	0	0
P_obscura	ObCe	ObCe5	root	root_5B	888	1348	670	253	0	0
P_obscura	ObCe	ObCe5	stem	stem	663	1480	583	314	41	0
P_obscura	ObCL	ObCL1	calyx	calyx	581	1494	112	0	0	0
P_obscura	ObCL	ObCL1	corolla	corolla	2220	2178	174	0	0	0
P_obscura	ObCL	ObCL1	leaf	leaf	1884	1638	252	0	0	0
P_obscura	ObCL	ObCL1	root	root_1A	413	1401	1369	360	0	0
P_obscura	ObCL	ObCL1	root	root_1B	1077	1519	1232	324	0	0
P_obscura	ObCL	ObCL1	root	root_1C	122	1023	1556	129	0	0
P_obscura	ObCL	ObCL1	root	root_1D	512	1115	752	52	0	0
P_obscura	ObCL	ObCL1	root	root_1E	535	906	612	55	0	0
P_obscura	ObCL	ObCL1	root	root_1F	504	1363	1659	156	0	0
P_obscura	ObCL	ObCL1	root	root_1G	662	1288	854	56	0	0
P_obscura	ObCL	ObCL1	stem	stem	963	1393	745	1216	35	0
P_obscura	ObCL	ObCL2	calyx	calyx	1260	1597	150	0	0	0
P_obscura	ObCL	ObCL2	corolla	corolla	2147	980	88	0	0	0
P_obscura	ObCL	ObCL2	leaf	leaf	1943	642	387	0	0	0

Species	*Locality*	*Plant*	*Organ*	*ORG*_rep	*2C*	*4C*	*8C*	*16C*	*32C*	*64C*
P_obscura	ObCL	ObCL2	root	root_2A	501	1030	1421	283	0	0
P_obscura	ObCL	ObCL2	root	root_2B	946	1232	1376	189	0	0
P_obscura	ObCL	ObCL2	root	root_2C	319	650	266	0	0	0
P_obscura	ObCL	ObCL2	root	root_2D	814	1347	1629	49	0	0
P_obscura	ObCL	ObCL2	stem	stem	1100	1468	820	430	0	0
P_obscura	ObCL	ObCL3	calyx	calyx	1757	1435	104	0	0	0
P_obscura	ObCL	ObCL3	corolla	corolla	2082	955	41	0	0	0
P_obscura	ObCL	ObCL3	leaf	leaf	2109	1854	383	0	0	0
P_obscura	ObCL	ObCL3	root	root_3A	1032	1677	526	0	0	0
P_obscura	ObCL	ObCL3	root	root_3B	638	1562	1709	351	0	0
P_obscura	ObCL	ObCL3	root	root_3C	385	871	853	75	0	0
P_obscura	ObCL	ObCL3	stem	stem	1057	1485	1281	157	0	0
P_obscura	ObCL	ObCL4	calyx	calyx	715	974	70	0	0	0
P_obscura	ObCL	ObCL4	corolla	corolla	2041	1640	64	0	0	0
P_obscura	ObCL	ObCL4	leaf	leaf	1752	1293	162	0	0	0
P_obscura	ObCL	ObCL4	root	root_4A	714	1305	480	8	0	0
P_obscura	ObCL	ObCL4	stem	stem	891	1425	780	627	0	0
P_obscura	ObCL	ObCL5	calyx	calyx	1453	1059	81	0	0	0
P_obscura	ObCL	ObCL5	corolla	corolla	3930	2644	113	0	0	0
P_obscura	ObCL	ObCL5	leaf	leaf	2451	1484	38	0	0	0
P_obscura	ObCL	ObCL5	root	root_5A	668	1804	1110	197	0	0
P_obscura	ObCL	ObCL5	root	root_5B	332	1308	1477	498	0	0
P_obscura	ObCL	ObCL5	root	root_5D	401	813	377	44	0	0
P_obscura	ObCL	ObCL5	stem	stem	915	1457	1068	105	0	0
P_obscura	ObDV	ObDV1	calyx	calyx	651	404	48	0	0	0
P_obscura	ObDV	ObDV1	corolla	corolla	1911	1558	57	0	0	0
P_obscura	ObDV	ObDV1	leaf	leaf	2177	1036	0	0	0	0
P_obscura	ObDV	ObDV1	root	root_1A	401	1301	1486	398	0	0
P_obscura	ObDV	ObDV1	stem	stem	730	1487	785	416	38	0
P_obscura	ObDV	ObDV2	calyx	calyx	1160	1474	96	0	0	0
P_obscura	ObDV	ObDV2	corolla	corolla	1715	1738	127	0	0	0
P_obscura	ObDV	ObDV2	leaf	leaf	2595	2057	445	0	0	0
P_obscura	ObDV	ObDV2	root	root_2B	275	1374	743	0	0	0
P_obscura	ObDV	ObDV2	stem	stem	832	1747	706	534	30	0
P_obscura	ObDV	ObDV3	calyx	calyx	1728	1113	75	18	0	0
P_obscura	ObDV	ObDV3	corolla	corolla	1733	1173	79	27	0	0
P_obscura	ObDV	ObDV3	leaf	leaf	2636	1225	1123	0	0	0
P_obscura	ObDV	ObDV3	root	root_3A	171	824	828	60	0	0
P_obscura	ObDV	ObDV3	root	root_3B	716	1381	1323	162	0	0
P_obscura	ObDV	ObDV3	root	root_3C	361	747	608	90	0	0
P_obscura	ObDV	ObDV3	root	root_3D	518	916	613	0	0	0
P_obscura	ObDV	ObDV3	stem	stem	756	1494	750	774	74	0
P_obscura	ObDV	ObDV4	calyx	calyx	833	647	34	10	0	0
P_obscura	ObDV	ObDV4	corolla	corolla	1652	1275	54	23	0	0

Species	*Locality*	*Plant*	*Organ*	*ORG*_rep	*2C*	*4C*	*8C*	*16C*	*32C*	*64C*
P_obscura	ObDV	ObDV4	leaf	leaf	3274	2044	450	0	0	0
P_obscura	ObDV	ObDV4	root	root_4A	704	1453	1113	138	0	0
P_obscura	ObDV	ObDV4	root	root_4B	238	1610	842	0	0	0
P_obscura	ObDV	ObDV4	stem	stem	779	1518	648	519	28	0
P_obscura	ObDV	ObDV5	calyx	calyx	1295	804	68	14	0	0
P_obscura	ObDV	ObDV5	corolla	corolla	2146	1496	55	14	0	0
P_obscura	ObDV	ObDV5	leaf	leaf	3148	1931	0	0	0	0
P_obscura	ObDV	ObDV5	root	root_5A	340	1655	977	0	0	0
P_obscura	ObDV	ObDV5	root	root_5B	307	1738	569	0	0	0
P_obscura	ObDV	ObDV5	root	root_5C	900	905	647	97	0	0
P_obscura	ObDV	ObDV5	stem	stem	1160	2199	1227	358	13	0
P_obscura	ObKa	ObKa1	calyx	calyx	738	1611	126	0	0	0
P_obscura	ObKa	ObKa1	corolla	corolla	2977	2357	100	0	0	0
P_obscura	ObKa	ObKa1	leaf	leaf	1676	985	63	0	0	0
P_obscura	ObKa	ObKa1	root	root_1A	435	1401	1429	216	0	0
P_obscura	ObKa	ObKa1	root	root_1B	462	857	592	164	0	0
P_obscura	ObKa	ObKa1	root	root_1C	644	1876	1697	233	0	0
P_obscura	ObKa	ObKa1	root	root_1D	478	1096	651	41	0	0
P_obscura	ObKa	ObKa1	stem	stem	849	1523	623	691	32	0
P_obscura	ObKa	ObKa2	calyx	calyx	835	1254	90	0	0	0
P_obscura	ObKa	ObKa2	corolla	corolla	1988	1942	136	0	0	0
P_obscura	ObKa	ObKa2	leaf	leaf	2093	1095	0	0	0	0
P_obscura	ObKa	ObKa2	root	root_2A	964	1458	352	0	0	0
P_obscura	ObKa	ObKa2	root	root_2B	823	1663	491	22	0	0
P_obscura	ObKa	ObKa2	stem	stem	577	1452	512	867	154	0
P_obscura	ObKa	ObKa3	calyx	calyx	2147	1517	73	0	0	0
P_obscura	ObKa	ObKa3	corolla	corolla	2008	1085	33	0	0	0
P_obscura	ObKa	ObKa3	leaf	leaf	2300	1074	612	0	0	0
P_obscura	ObKa	ObKa3	root	root_3B	464	724	314	0	0	0
P_obscura	ObKa	ObKa3	root	root_3C	487	702	229	0	0	0
P_obscura	ObKa	ObKa3	stem	stem	341	1503	942	199	0	0
P_obscura	ObKa	ObKa4	calyx	calyx	942	1556	62	0	0	0
P_obscura	ObKa	ObKa4	corolla	corolla	1978	1578	112	0	0	0
P_obscura	ObKa	ObKa4	leaf	leaf	1875	971	56	0	0	0
P_obscura	ObKa	ObKa4	root	root_4A	657	1788	1456	55	0	0
P_obscura	ObKa	ObKa4	root	root_4B	312	746	1710	191	0	0
P_obscura	ObKa	ObKa4	root	root_4C	208	1584	1005	0	0	0
P_obscura	ObKa	ObKa4	stem	stem	765	1564	833	635	0	0
P_obscura	ObKa	ObKa5	calyx	calyx	2234	1915	189	14	0	0
P_obscura	ObKa	ObKa5	corolla	corolla	490	2087	127	0	0	0
P_obscura	ObKa	ObKa5	leaf	leaf	1630	943	27	0	0	0
P_obscura	ObKa	ObKa5	root	root_5A	593	1248	1006	142	0	0
P_obscura	ObKa	ObKa5	root	root_5B	86	1095	1541	73	0	0
P_obscura	ObKa	ObKa5	root	root_5C	258	1610	1341	78	0	0

Species	*Locality*	*Plant*	*Organ*	*ORG*_rep	*2C*	*4C*	*8C*	*16C*	*32C*	*64C*
P_obscura	ObKa	ObKa5	root	root_5D	445	1126	1586	232	0	0
P_obscura	ObKa	ObKa5	root	root_5E	372	1040	870	54	0	0
P_obscura	ObKa	ObKa5	stem	stem	412	1617	665	752	0	0
P_officinalis	OfHT	OfHT1	calyx	calyx	1747	428	32	0	0	0
P_officinalis	OfHT	OfHT1	corolla	corolla	4051	1425	55	0	0	0
P_officinalis	OfHT	OfHT1	root	root_1A	1668	1062	126	0	0	0
P_officinalis	OfHT	OfHT1	root	root_1B	1493	1342	183	0	0	0
P_officinalis	OfHT	OfHT1	stem	stem	1533	1528	1043	156	0	0
P_officinalis	OfHT	OfHT2	calyx	calyx	2842	1043	49	0	0	0
P_officinalis	OfHT	OfHT2	corolla	corolla	2470	470	13	0	0	0
P_officinalis	OfHT	OfHT2	root	root_2A	1278	1174	558	29	0	0
P_officinalis	OfHT	OfHT2	root	root_2B	1271	927	575	49	0	0
P_officinalis	OfHT	OfHT2	root	root_2C	1446	1155	46	34	0	0
P_officinalis	OfHT	OfHT2	root	root_2D	1264	872	339	0	0	0
P_officinalis	OfHT	OfHT2	stem	stem	1233	1277	926	525	0	0
P_officinalis	OfHT	OfHT3	calyx	calyx	2725	943	109	0	0	0
P_officinalis	OfHT	OfHT3	corolla	corolla	2776	617	20	0	0	0
P_officinalis	OfHT	OfHT3	root	root_3A	1096	1429	532	0	0	0
P_officinalis	OfHT	OfHT3	root	root_3B	853	943	57	0	0	0
P_officinalis	OfHT	OfHT3	root	root_3C	1627	1785	381	0	0	0
P_officinalis	OfHT	OfHT3	root	root_3D	1447	1029	143	0	0	0
P_officinalis	OfHT	OfHT3	root	root_3E	1090	726	124	0	0	0
P_officinalis	OfHT	OfHT3	stem	stem	1140	1432	861	760	0	0
P_officinalis	OfHT	OfHT4	calyx	calyx	3003	723	55	0	0	0
P_officinalis	OfHT	OfHT4	corolla	corolla	4909	1465	120	0	0	0
P_officinalis	OfHT	OfHT4	root	root_4A	908	1435	691	0	0	0
P_officinalis	OfHT	OfHT4	root	root_4B	846	1581	322	0	0	0
P_officinalis	OfHT	OfHT4	root	root_4C	961	1518	987	0	0	0
P_officinalis	OfHT	OfHT4	root	root_4D	858	1570	289	0	0	0
P_officinalis	OfHT	OfHT4	stem	stem	1153	1483	726	580	9	0
P_officinalis	OfHT	OfHT5	calyx	calyx	2816	1005	84	0	0	0
P_officinalis	OfHT	OfHT5	corolla	corolla	2173	857	44	0	0	0
P_officinalis	OfHT	OfHT5	root	root_5A	762	1676	847	0	0	0
P_officinalis	OfHT	OfHT5	root	root_5B	981	1475	576	0	0	0
P_officinalis	OfHT	OfHT5	root	root_5C	672	1448	656	0	0	0
P_officinalis	OfHT	OfHT5	root	root_5D	1085	1513	330	0	0	0
P_officinalis	OfHT	OfHT5	root	root_5E	1536	1798	1019	0	0	0
P_officinalis	OfHT	OfHT5	stem	stem	1158	1616	802	676	131	0
P_officinalis	OfLe	OfLe1	calyx	calyx	2929	479	53	0	0	0
P_officinalis	OfLe	OfLe1	corolla	corolla	2229	403	31	0	0	0
P_officinalis	OfLe	OfLe1	root	root_1A	1292	1536	235	0	0	0
P_officinalis	OfLe	OfLe1	root	root_1B	376	1419	466	0	0	0
P_officinalis	OfLe	OfLe1	root	root_1C	1082	1375	1549	0	0	0
P_officinalis	OfLe	OfLe1	root	root_1D	619	1476	280	0	0	0

Species	*Locality*	*Plant*	*Organ*	*ORG*_rep	*2C*	*4C*	*8C*	*16C*	*32C*	*64C*
P_officinalis	OfLe	OfLe1	stem	stem	1063	1454	667	308	0	0
P_officinalis	OfLe	OfLe2	calyx	calyx	1675	491	19	0	0	0
P_officinalis	OfLe	OfLe2	corolla	corolla	2182	366	15	0	0	0
P_officinalis	OfLe	OfLe2	root	root_2A	653	1221	1046	0	0	0
P_officinalis	OfLe	OfLe2	root	root_2B	500	1342	1403	22	0	0
P_officinalis	OfLe	OfLe2	root	root_2C	919	1368	808	18	0	0
P_officinalis	OfLe	OfLe2	root	root_2D	786	822	621	0	0	0
P_officinalis	OfLe	OfLe2	stem	stem	1265	1468	703	728	70	0
P_officinalis	OfLe	OfLe3	calyx	calyx	1540	422	36	0	0	0
P_officinalis	OfLe	OfLe3	corolla	corolla	3725	832	0	0	0	0
P_officinalis	OfLe	OfLe3	root	root_3A	904	976	178	0	0	0
P_officinalis	OfLe	OfLe3	root	root_3B	1127	1250	376	0	0	0
P_officinalis	OfLe	OfLe3	root	root_3C	1359	1700	308	0	0	0
P_officinalis	OfLe	OfLe3	root	root_3D	912	1496	419	0	0	0
P_officinalis	OfLe	OfLe3	root	root_3E	985	1280	474	0	0	0
P_officinalis	OfLe	OfLe3	stem	stem	1099	1470	676	690	247	0
P_officinalis	OfLe	OfLe4	calyx	calyx	2191	425	23	0	0	0
P_officinalis	OfLe	OfLe4	corolla	corolla	2786	494	25	0	0	0
P_officinalis	OfLe	OfLe4	root	root_4A	386	1853	609	0	0	0
P_officinalis	OfLe	OfLe4	root	root_4B	708	1504	675	0	0	0
P_officinalis	OfLe	OfLe4	root	root_4C	706	1632	773	0	0	0
P_officinalis	OfLe	OfLe4	root	root_4D	848	1488	918	0	0	0
P_officinalis	OfLe	OfLe4	root	root_4E	1216	1592	689	0	0	0
P_officinalis	OfLe	OfLe4	stem	stem	1259	1577	637	760	0	0
P_officinalis	OfLe	OfLe5	calyx	calyx	957	319	0	0	0	0
P_officinalis	OfLe	OfLe5	corolla	corolla	2427	466	0	0	0	0
P_officinalis	OfLe	OfLe5	root	root_5A	1140	2031	1809	80	0	0
P_officinalis	OfLe	OfLe5	root	root_5B	1127	1472	826	86	0	0
P_officinalis	OfLe	OfLe5	root	root_5C	1056	1316	1089	92	0	0
P_officinalis	OfLe	OfLe5	root	root_5E	778	1714	357	0	0	0
P_officinalis	OfLe	OfLe5	stem	stem	1285	1484	585	607	62	0
P_officinalis	OfNi	OfNi1	calyx	calyx	2575	365	0	0	0	0
P_officinalis	OfNi	OfNi1	corolla	corolla	2111	393	0	0	0	0
P_officinalis	OfNi	OfNi1	root	root_1A	505	1808	1513	0	0	0
P_officinalis	OfNi	OfNi1	root	root_1B	252	1819	357	0	0	0
P_officinalis	OfNi	OfNi1	root	root_1C	196	1794	1171	0	0	0
P_officinalis	OfNi	OfNi1	root	root_1D	846	1411	1569	59	0	0
P_officinalis	OfNi	OfNi1	root	root_1E	1617	1665	222	0	0	0
P_officinalis	OfNi	OfNi1	stem	stem	1290	1519	746	625	0	0
P_officinalis	OfNi	OfNi2	calyx	calyx	1208	252	0	0	0	0
P_officinalis	OfNi	OfNi2	corolla	corolla	2907	1050	47	0	0	0
P_officinalis	OfNi	OfNi2	root	root_2A	785	1270	1691	154	0	0
P_officinalis	OfNi	OfNi2	root	root_2B	748	1272	985	0	0	0
P_officinalis	OfNi	OfNi2	root	root_2C	760	1557	418	0	0	0

Species	*Locality*	*Plant*	*Organ*	*ORG*_rep	*2C*	*4C*	*8C*	*16C*	*32C*	*64C*
P_officinalis	OfNi	OfNi2	root	root_2D	1376	1411	657	33	0	0
P_officinalis	OfNi	OfNi2	root	root_2E	170	1789	1127	0	0	0
P_officinalis	OfNi	OfNi2	stem	stem	778	1675	844	646	76	0
P_officinalis	OfNi	OfNi3	calyx	calyx	3125	480	41	0	0	0
P_officinalis	OfNi	OfNi3	corolla	corolla	1855	349	29	0	0	0
P_officinalis	OfNi	OfNi3	root	root_3A	927	1554	804	31	0	0
P_officinalis	OfNi	OfNi3	root	root_3B	1382	1536	561	0	0	0
P_officinalis	OfNi	OfNi3	root	root_3C	1012	1535	428	0	0	0
P_officinalis	OfNi	OfNi3	root	root_3E	986	1267	336	0	0	0
P_officinalis	OfNi	OfNi3	stem	stem	1549	1906	951	632	78	0
P_officinalis	OfNi	OfNi4	calyx	calyx	1590	154	20	0	0	0
P_officinalis	OfNi	OfNi4	corolla	corolla	2020	402	0	0	0	0
P_officinalis	OfNi	OfNi4	root	root_4A	723	2765	1276	0	0	0
P_officinalis	OfNi	OfNi4	root	root_4B	810	1462	847	0	0	0
P_officinalis	OfNi	OfNi4	root	root_4C	416	1683	1108	0	0	0
P_officinalis	OfNi	OfNi4	root	root_4D	541	1515	563	0	0	0
P_officinalis	OfNi	OfNi4	root	root_4E	1020	1541	382	0	0	0
P_officinalis	OfNi	OfNi4	stem	stem	714	1377	631	658	78	0
P_officinalis	OfNi	OfNi5	calyx	calyx	1574	221	0	0	0	0
P_officinalis	OfNi	OfNi5	corolla	corolla	2003	670	46	0	0	0
P_officinalis	OfNi	OfNi5	root	root	483	1671	284	0	0	0
P_officinalis	OfNi	OfNi5	stem	stem	1206	1925	625	580	82	0
P_officinalis	OfSm	OfSm1	calyx	calyx	2269	625	26	0	0	0
P_officinalis	OfSm	OfSm1	corolla	corolla	2119	770	29	0	0	0
P_officinalis	OfSm	OfSm1	root	root_1A	865	1198	416	0	0	0
P_officinalis	OfSm	OfSm1	root	root_1B	496	1478	515	0	0	0
P_officinalis	OfSm	OfSm1	root	root_1C	1252	1624	424	0	0	0
P_officinalis	OfSm	OfSm1	root	root_1D	1096	1399	424	0	0	0
P_officinalis	OfSm	OfSm1	root	root_1E	1441	1086	274	0	0	0
P_officinalis	OfSm	OfSm1	stem	stem	892	1380	767	444	11	0
P_officinalis	OfSm	OfSm2	calyx	calyx	1825	361	0	0	0	0
P_officinalis	OfSm	OfSm2	corolla	corolla	2466	536	0	0	0	0
P_officinalis	OfSm	OfSm2	root	root_2A	1072	3045	155	0	0	0
P_officinalis	OfSm	OfSm2	root	root_2B	1461	1336	399	0	0	0
P_officinalis	OfSm	OfSm2	stem	stem	1429	1484	1023	464	0	0
P_officinalis	OfSm	OfSm3	calyx	calyx	1671	206	0	0	0	0
P_officinalis	OfSm	OfSm3	corolla	corolla	2352	458	0	0	0	0
P_officinalis	OfSm	OfSm3	root	root_3A	958	1488	1683	57	0	0
P_officinalis	OfSm	OfSm3	root	root_3B	1680	975	1467	101	0	0
P_officinalis	OfSm	OfSm3	root	root_3C	1138	1505	535	0	0	0
P_officinalis	OfSm	OfSm3	stem	stem	1183	1395	783	174	0	0
P_officinalis	OfSm	OfSm4	calyx	calyx	1621	223	0	0	0	0
P_officinalis	OfSm	OfSm4	corolla	corolla	3978	645	0	0	0	0
P_officinalis	OfSm	OfSm4	root	root_4A	530	1589	792	0	0	0

Species	*Locality*	*Plant*	*Organ*	*ORG*_rep	*2C*	*4C*	*8C*	*16C*	*32C*	*64C*
P_officinalis	OfSm	OfSm4	root	root_4B	1112	1515	890	0	0	0
P_officinalis	OfSm	OfSm4	root	root_4C	801	1585	901	0	0	0
P_officinalis	OfSm	OfSm4	root	root_4D	1087	1641	837	0	0	0
P_officinalis	OfSm	OfSm4	stem	stem	852	1565	826	503	0	0
P_officinalis	OfSm	OfSm5	calyx	calyx	2527	807	0	0	0	0
P_officinalis	OfSm	OfSm5	corolla	corolla	2022	594	20	0	0	0
P_officinalis	OfSm	OfSm5	root	root_5A	941	1555	920	0	0	0
P_officinalis	OfSm	OfSm5	root	root_5B	1077	1026	104	0	0	0
P_officinalis	OfSm	OfSm5	root	root_5C	458	1612	627	0	0	0
P_officinalis	OfSm	OfSm5	root	root_5D	757	890	973	0	0	0
P_officinalis	OfSm	OfSm5	root	root_5E	1970	1722	269	0	0	0
P_officinalis	OfSm	OfSm5	stem	stem	1721	1773	572	0	0	0

SUPPORTING INFORMATION Figure S1

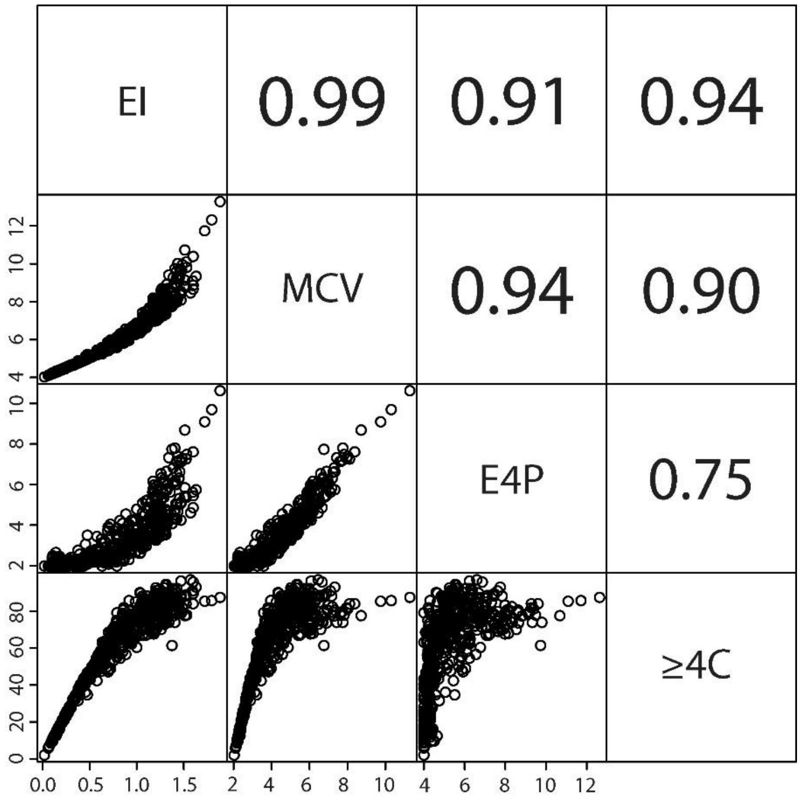

Figure S1 Correlation analyses of endopolyploidy parameters, endoreduplication index (EI), mean C value (MCV), mean ploidy level of endopolyploid nuclei (E4P) and proportion of cells with > 2C level (≥4C) calculated based on 530 FCM records. Spearman correlation coefficients significant at $p < 0.001$ are reported.

SUPPORTING INFORMATION Figure S2

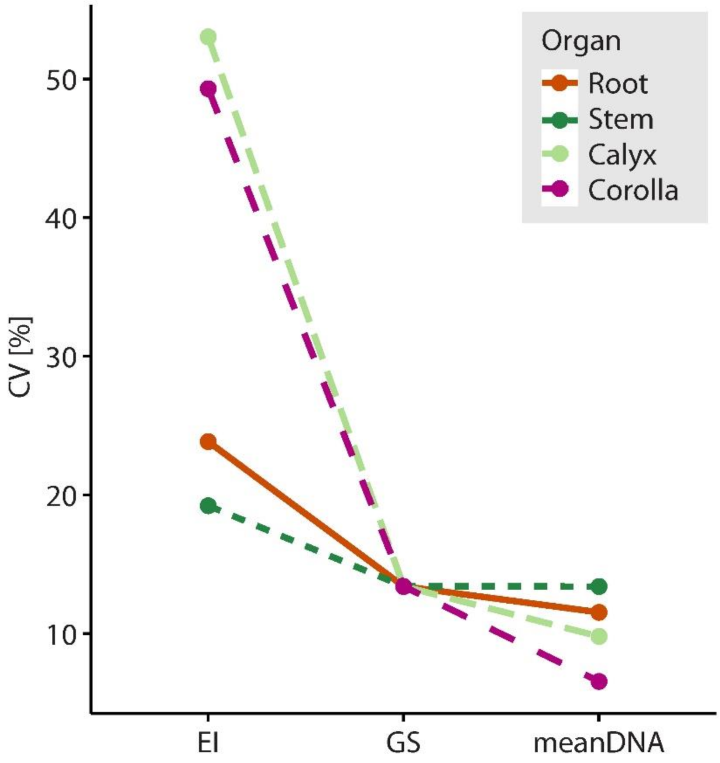

Figure S2 Comparison of variation (expressed as mean coefficient of variation, CV) of endopolyploidy level (expressed as EI), genome size and tissue-specific mean DNA content (meanDNA) in four species of *Pulmonaria*, *P. mollis* (2*n* = 18), *P. murinii* (2*n* = 14), *P. obscura* (2*n* = 14), and *P. officinalis* (2*n* = 16) for four investigated organs, root, stem, calyx and corolla.
